# Improved Health Outcomes from Hepatitis C Treatment Scale-Up in Spain’s Prisons: A Cost-Effectiveness Study

**DOI:** 10.1038/s41598-019-52564-0

**Published:** 2019-11-14

**Authors:** Ozden O. Dalgic, Sumeyye Samur, Anne C. Spaulding, Susana Llerena, Carmen Cobo, Turgay Ayer, Mark S. Roberts, Javier Crespo, Jagpreet Chhatwal

**Affiliations:** 1 Institute for Technology Assessment, Massachusetts General Hospital, Harvard Medical School, Boston, Massachusetts USA; 2 Department of Epidemiology, Rollins School of Public Health, Emory University, Atlanta, Georgia USA; 3 Department of Gastroenterology and Hepatology, Marques de Valdecilla University Hospital, Santander, Spain; 4 Medical Service, El Dueso Penitentiary Centre, Santoña, Spain; 5 H. Milton Stewart School of Industrial and Systems Engineering, Georgia Institute of Technology, Atlanta, Georgia USA; 6 Department of Health Policy and Management, University of Pittsburgh, Pittsburgh, Pennsylvania USA

**Keywords:** Hepatitis C, Health care economics, Health policy, Epidemiology, Population screening

## Abstract

Hepatitis C virus (HCV) is 15 times more prevalent among persons in Spain’s prisons than in the community. Recently, Spain initiated a pilot program, JAILFREE-C, to treat HCV in prisons using direct-acting antivirals (DAAs). Our aim was to identify a cost-effective strategy to scale-up HCV treatment in all prisons. Using a validated agent-based model, we simulated the HCV landscape in Spain’s prisons considering disease transmission, screening, treatment, and prison-community dynamics. Costs and disease outcomes under status quo were compared with strategies to scale-up treatment in prisons considering prioritization (HCV fibrosis stage vs. HCV prevalence of prisons), treatment capacity (2,000/year vs. unlimited) and treatment initiation based on sentence lengths (>6 months vs. any). Scaling-up treatment by treating all incarcerated persons irrespective of their sentence length provided maximum health benefits–preventing 10,200 new cases of HCV, and 8,300 HCV-related deaths between 2019–2050; 90% deaths prevented would have occurred in the community. Compared with status quo, this strategy increased quality-adjusted life year (QALYs) by 69,700 and costs by €670 million, yielding an incremental cost-effectiveness ratio of €9,600/QALY. Scaling-up HCV treatment with DAAs for the entire Spanish prison population, irrespective of sentence length, is cost-effective and would reduce HCV burden.

## Introduction

Hepatitis C virus (HCV) infection is 10–15 times more prevalent among persons in prisons in Spain compared with the general population^1^. If untreated, HCV can lead to cirrhosis, hepatocellular carcinoma and death. Injection drug use is the most common mode of HCV transmission in Spain, and people who inject drugs (PWID) account for 20–55% of persons imprisoned^2^. Eventually, the majority of incarcerated persons are released back into society. A practice of releasing before treatment can substantially contribute to HCV spread in society.

The recent availability of direct-acting antivirals (DAAs) offers an unprecedented opportunity to cure HCV. Compared to older treatments, these antivirals are superior — their efficacy is greater than 95% in most patient groups, treatment courses (8–12 weeks) are short, and the side effect profile is benign^3^. In addition, timely treatment of HCV in high-risk groups such as in PWIDs can reduce HCV transmission, both in prisons and outside community^4,5^. However, a vast majority of persons living with HCV in Spain have limited access to DAAs.

In 2016, Spain initiated an innovative pilot program to screen and treat HCV in prisons^2^. A clinical trial, JAILFREE-C study, evaluated the performance of systematic screening and treatment for HCV among those residing in El Dueso — a long-stay prison. Results of the study showed that sustained virologic response (SVR), a surrogate for cure, was achieved in more than 95% of treated patients.

There is a need for and interest in scaling-up HCV treatment in all existing prison facilities in Spain. However, the cost-effectiveness and long-term impact of different strategies to scale-up HCV treatment are not known, and several important questions remain unanswered. For example, should certain prisons get priority for treatment over other prisons (e.g. based on HCV prevalence); should certain inmates get priority over other inmates (e.g., based on disease severity) within each prison; and what should be the treatment capacity? Therefore, the objective of our study was to identify a cost-effective strategy to scale-up HCV treatment in all of Spain’s prisons, and project the long-term clinical and economic benefits of treating inmates, who may face re-exposure to HCV.

## Methods

### Model overview

**Figure 1:**
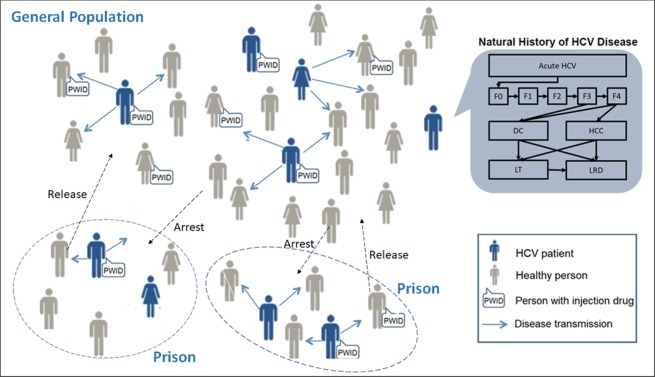
Model schematic showing the prison population and general population and the dynamic movement between the two groups (dashed arrows). Individuals are defined by demographic characteristics, liver disease stage, and injection drug use (IDU) status. Throughout the simulation, several characteristics are updated: age, IDU status, HCV infection status (infected individuals shown in blue), liver disease stage, and location (inside or outside prison). HCV-infected individuals can transmit disease to others in their immediate network (solid arrows). The natural history of chronic HCV disease is represented by Markov states (top right inset). Stages of chronic HCV disease are defined by METAVIR fibrosis scores. Advanced liver diseases stages are DC, HCC, LT, and LRDs. DC = decompensated cirrhosis; F0 = no fibrosis; F1 = portal fibrosis without septa; F2 = portal fibrosis with few septa; F3 = numerous septa without cirrhosis; F4 = compensated cirrhosis; HCC = hepatocellular carcinoma; HCV = hepatitis C virus; IDU = injection drug use; LRD = liver-related death; LT = liver transplantation.

We adapted a previously-developed and validated agent-based simulation model — treatment as prevention of HCV (*TapHCV model*), to simulate HCV epidemic in Spain’s prisons as well as in the general population^4^. TapHCV model included dynamic movement of people in and out of prisons, HCV transmission between PWID, the natural history of HCV based on a previously validated Markov model^6^, and treatment with oral DAAs (Fig. 1). The model was developed in C++ programming language from national healthcare payer’s perspective with a 31-year time horizon using monthly cycles. The model evaluated the long-term clinical benefits and costs of different strategies to scale-up HCV treatment in Spain’s prisons.

### Baseline population

**Figure 2:**
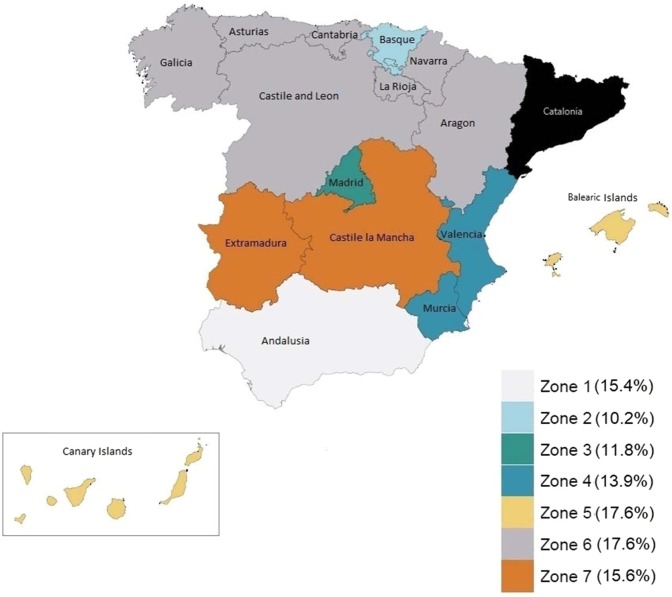
Spain’s regions were divided in seven zones shown by different colors. Numbers in the parenthesis correspond to HCV prevalence in each zone. The region shaded in black was not included.

The baseline population in the *TapHCV* model represented Spain’s population (46.56 million), both in the community and prisons, starting from year 2015 onwards. For modeling purposes, we divided Spain’s correctional facilities into seven geographical “virtual” zones consisting of a total of 41,020 inmates (96.2% were males), such that each zone had approximately equal number of persons incarcerated (Fig. 2)^2,7^. These zones were created for the purpose of reducing simulation noise in model outcomes for prisons having smaller population size. We estimated HCV prevalence in each facility using data reported by Spain’s Ministry of Interior (Table S1)^1^. We estimated distribution of HCV genotypes commonly observed in Spain (genotypes 1, 2, 3 and 4), chronic HCV fibrosis stages defined by METAVIR (Meta-Analysis of Histologic Data in Viral Hepatitis) fibrosis scores, i.e. F0–F4, and treatment history (previously treated or treatment-naïve) using published studies (Table 1).

**Table 1:** Baseline population characteristics and model parameters used in TapHCV model for Spain.

Model Parameter	Value		Range
Population			
General population	46,560,000		—
Inmates^1^	41,020		—
Gender (male %)			
General population^23^	49%		—
Prisons^2^	96.2%		—
Transmission probability^4^	0.000255		0.000085–0.000425
Self-clearance probability^24^	0.25		0.23–0.28
Newborn infection rate^25^	0.0093%		0.0061%–0.018%
PWID–non-PWID interaction probability^4^	0.012		—
Status quo treatment capacity in the prisons	160 (calibrated)		—
Prevalence of PWID			
In prisons (Active PWID^2^; Former PWID^26,27^)	37.2%; 20.5%		—
Outside of prisons (Active PWID^28^; Former PWID^26,27^)	0.03%; 1.3%		—
Birth-rate per 1,000 population (annual)^23^	15		—
Standardized mortality ratio (SMR)			
PWID^29^	2.54		—
Inmates^10^	0.85		—
HCV genotype^2,8,30–36^ [Genotype 1; Genotype 2; Genotype 3; Genotype 4]	[50.2%; 2.37%; 27.8%; 19.7%]		—
Chronic hepatitis C disease stage distribution^2,8,30–36^			
[F0; F1; F2; F3; F4; DC; HCC]	[14.2%; 31.6%; 16.5%; 14.1%; 20.4%; 3.09%; 0.29%]		—
Proportion of patients aware of their HCV status			
In general population^9^	30%		0.7–0.9
In prisons^8^	80%		0.05–0.55
Proportion of treatment-experienced patients in prisons^2^	23.1%		0.131–0.331
Agent’s behavior^6^			
F0 HCV diagnosis probability (annual)	0.037		0.028–0.046
F1 HCV diagnosis probability (annual)	0.030		0.022–0.037
F2 HCV diagnosis probability (annual)	0.042		0.032–0.052
F3 HCV diagnosis probability (annual)	0.046		0.035–0.057
F4 HCV diagnosis probability (annual)	0.163		0.124–0.199
Aware reduction factor	0.5		0.25–0.75
Treatment reduction factor	0.0		0.0–1.0
Transition probabilities (annual)			
F0 to F1^37^	${e}^{-2.0124-(0.07589\times duration)+(0.3247\times 0.5)+(0.5063\times f(male))+(0.4839\times f(G1))}\,$		
F1 to F2^37^	${e}^{-1.5387-(0.06146\times duration)+(0.8001\times f({\text{excess alcohol}}))}$		
F2 to F3^37^	${e}^{-1.6038+(0.0172\times {\text{age at HCV}})-(0.05939\times duration)+(0.4539\times 0.19)}$		
F3 to F4^37^	${e}^{-2.2898+(0.01689\times {\text{age at HCV}})-(0.03694\times duration)+(0.5963\times f(PWID))+(1.1682\times 0.31)-(0.4652\times f(G1))}$		
F3 to HCC^38^	0.008		0.003–0.014
F4 to DC^39^	0.039		0.01–0.079
F4 to HCC^39^	0.014		0.01–0.079
SVR after cirrhosis to DC^24^	0.008		0.002–0.036
SVR after cirrhosis to HCC^24^	0.005		0.002–0.013
DC to HCC^40^	0.068		0.03–0.083
DC to LT^41,42^	0.023		0.01–0.062
DC (first year) to LRD^40^	0.182		0.065–0.19
DC (subsequent year) to LRD^40^	0.112		0.065–0.19
HCC to LT^43,44^	0.040		0–0.14
HCC to LRD^39^	0.427		0.33–0.86
LT (first year) to LRD^45^	0.116		0.06–0.42
LT (subsequent year) to LRD^45^	0.044		0.024–0.11
Health state costs (annual) (€)^31^			
F0, F1	365		182.5–547.5
F2, F3	280		140–420
F4	560		280–840
DC	2,280		1,140–3,420
HCC	6,700		3,350–10,050
LT, first year	104,000		52,000–156,000
LT, subsequent year	17,800		89,00–267,00
Testing cost (one-time) (€)^46^			
HCV ELISA test (anti-HCV antibody test)	3		1.5–4.5
Quantitative HCV RNA	40		20–60
Fibroscan test	60		30–90
HCV treatment costs (one-time) (€)^32,33^	17,126		3,333–12,083
Health-related quality-of-life weights^34^			
F0, F1	0.93		0.99–0.837
F2, F3	0.93		0.99–0.837
F4	0.9		0.99–0.81
DC	0.8		0.88–0.72
HCC	0.79		0.869–0.711
First-year post-LT	0.84		0.924–0.756
Post SVR (F0-F1)	1		0.99–0.9
Post SVR (F2-F4)	0.93		0.99–0.837
Age-related quality-of-life weights^35^			
Age group	Male	Female	
0–29	0.928	0.913	—
30–39	0.918	0.893	—
40–49	0.887	0.863	—
50–59	0.861	0.837	—
60–69	0.84	0.811	—

f(male) = 1, if patient is male; and 0 if patient is female.

f(G1) = 1, if patient has hepatitis C virus (HCV) genotype 1; and 0 otherwise.

f(excess alcohol) = 1, if patients has excess alcohol consumption; and 0 otherwise. The prevalence of excess alcohol consumption was 24% for male inmates, 17% for female inmates, and 23% for general population.

f(PWID) = 1, if patients are active injection drug users; and 0 otherwise.

Abbreviations: HCV; hepatitis C virus; PWID, person who injects drug; SVR, sustained virology response; METAVIR, meta-analysis of histologic data in viral hepatitis; F0–F4, METAVIR fibrosis score. DC, decompensated cirrhosis; HCC, hepatocellular carcinoma; ELISA, enzyme-linked immunosorbant analysis; RNA, ribonucleic acid.

### HCV transmission and progression

We modeled HCV transmission separately in prisons and community. The transmission of HCV was resulted from a contact between an infected individual and a susceptible individual. In the community, infected individuals could contact anyone in the community, whereas in prisons, infected persons were able to contact susceptibles only within the same zone. During each cycle (i.e., month), an infected individual contacted a randomly-selected susceptible individual and could transmit the virus. We assumed PWIDs were more likely to be in contact with other PWIDs (Table 1). Similarly, non-PWIDs had a higher chance of contacting non-PWIDs. Once a contact was established between an infected and a susceptible individual, the virus could be transmitted with a certain probability (Table 1). The probability of transmission was halved if the infected individual was aware of his/her disease condition and this assumption was tested in sensitivity analysis (‘Aware reduction factor’ in Table 1). Treated individuals were susceptible to reinfection.

We defined the natural history of HCV using Markov health states, which was based on our previously published model (Table 1)^6^. All new infections started in an acute phase of HCV, which could either resolve by itself or develop into a chronic phase, defined by METAVIR fibrosis score F0. Patient’s fibrosis stage could progress from F0 to F4 (Fig. 1). Patients with F3 or F4 could develop decompensated cirrhosis, hepatocellular carcinoma. Patients with decompensated cirrhosis or hepatocellular carcinoma could receive a liver transplant or die because a liver-related mortality. All patients could also die because of a background mortality adjusted by their age, sex and injection drug use status.

### HCV awareness and screening

Because of high screening rates in the Spain prisons, 80% of the HCV-infected persons were aware of their disease^8^. In the community, only 30% of the infected patients were aware of their disease status^9^. Unaware patients could be diagnosed through HCV testing that included HCV antibody and HCV RNA tests. The one-step diagnosis using reflex HCV RNA testing is implemented in almost all of the Spanish penitentiary institutions.

### HCV treatment strategies

In the status quo, we calibrated the number of people initiating HCV treatments in Spain’s prisons using data from the Ministry of Internal Affairs reports^1^. We compared status quo — limited access to HCV treatment — with different treatment scale-up strategies with DAAs considering constrained and unlimited capacities. Specifically, we simulated the following four strategies: *Strategy 1* prioritizes *inmates* by their fibrosis stages (fibrosis scores F4, F3, F2, F1, and F0) with a treatment capacity of 2,000/year, irrespective of the prison or region. *Strategy 2* prioritizes *prisons* by their HCV prevalence with a treatment capacity of 2,000/year, irrespective of fibrosis stages. The 2,000/year capacity was chosen so that at most one third of the people infected with HCV could receive the treatment in a year. *Strategy 3* considers unlimited capacity. In *Strategies 1–3*, only those sentenced with more than six months are eligible for treatment. *Strategy 4* considers unlimited treatment capacity and assumed everyone, irrespective of their sentence length, is eligible for treatment. Because, all available oral DAAs have high efficacy, our analysis was relevant to all DAAs.

### Admission and release of prisoners

We simulated movement of people from the community to prisons and vice versa (Fig. 1). The baseline prison population and the lengths of sentence were estimated from published reports (Tables S1 and S2)^10^. The probability of incarceration was back-calculated such that average age of inmates and gender distribution, prevalence of PWIDs and former PWIDs in prisons, and prevalence of HCV antibody in prisons remained stable over time. See Supplementary Section 1 for the calculation of the incarceration probability.

### Costs and utilities

Our model included the cost of HCV testing, i.e. HCV antibody, HCV RNA, Fibroscan, antiviral treatment, and management of chronic HCV disease. HCV disease management costs included the cost associated with chronic HCV infection, decompensated cirrhosis, hepatocellular carcinoma and liver transplant. In the base case, we assumed the cost of DAA treatment was €17,126 in 2019 which was calculated from the total cost of all patients with HCV treated with sofosbuvir/ledipasvir between April 2015 and Sept 2018 divided by the number of patients treated with the new therapies in the same period^11,12^. We estimated HCV testing and disease management costs from published sources (Table 1)^13^.

To each individual in our model, we assigned health-related quality-of-life (QOL) weights, with 0 denoting death and 1 denoting perfect health, and adjusted them by IDU, age and sex (Table 1)^14^. We assumed the QOL of patients who achieved sustained virologic response were equivalent to that of the non-HCV infected individuals^15^.

### Model outcomes

For each strategy — status quo and four treatment scale-up strategies, we projected HCV-related deaths, decompensated cirrhosis and hepatocellular carcinoma until 2050. Further, for each strategy, we estimated total quality-adjusted life years (QALYs), total costs that included the cost of HCV screening, cost of antiviral treatment and the cost associated with HCV disease and its sequelae. We then estimated the incremental cost-effectiveness ratios (ICERs) of all treatment scale-up strategies. We also estimated annual HCV–associated cost in Spain’s prisons under status quo and different strategies for scaling-up treatment to all prisons. We estimated those outcomes from a national healthcare payer perspective, which included both prisons and the general community.

We also estimated *prison-related* HCV transmission and deaths, which resulted from inmates whose HCV was not treated while they were incarcerated. Some of these untreated inmates developed advanced HCV sequelae and/or infected others over time, inside and/or outside the prisons.

We performed one-way and two-way sensitivity analyses on key model parameters. For all outcomes, we ran our model 100 times (Monte Carlo runs) to reduce first-order uncertainty (i.e. simulation noise).

## Results

### Model validation

The TapHCV model closely matched HCV prevalence in Spain’s prisons from 2010–2015 as reported by the Ministry Interior reports (Fig. S1)^1^. Because the current treatment rate and those of previous years in prisons were not publicly available, we estimated the annual treatment rate under status quo from 2010–2018 through calibration to be equal to 160 persons per year.

### Cost-effectiveness analysis

**Figure 3:**
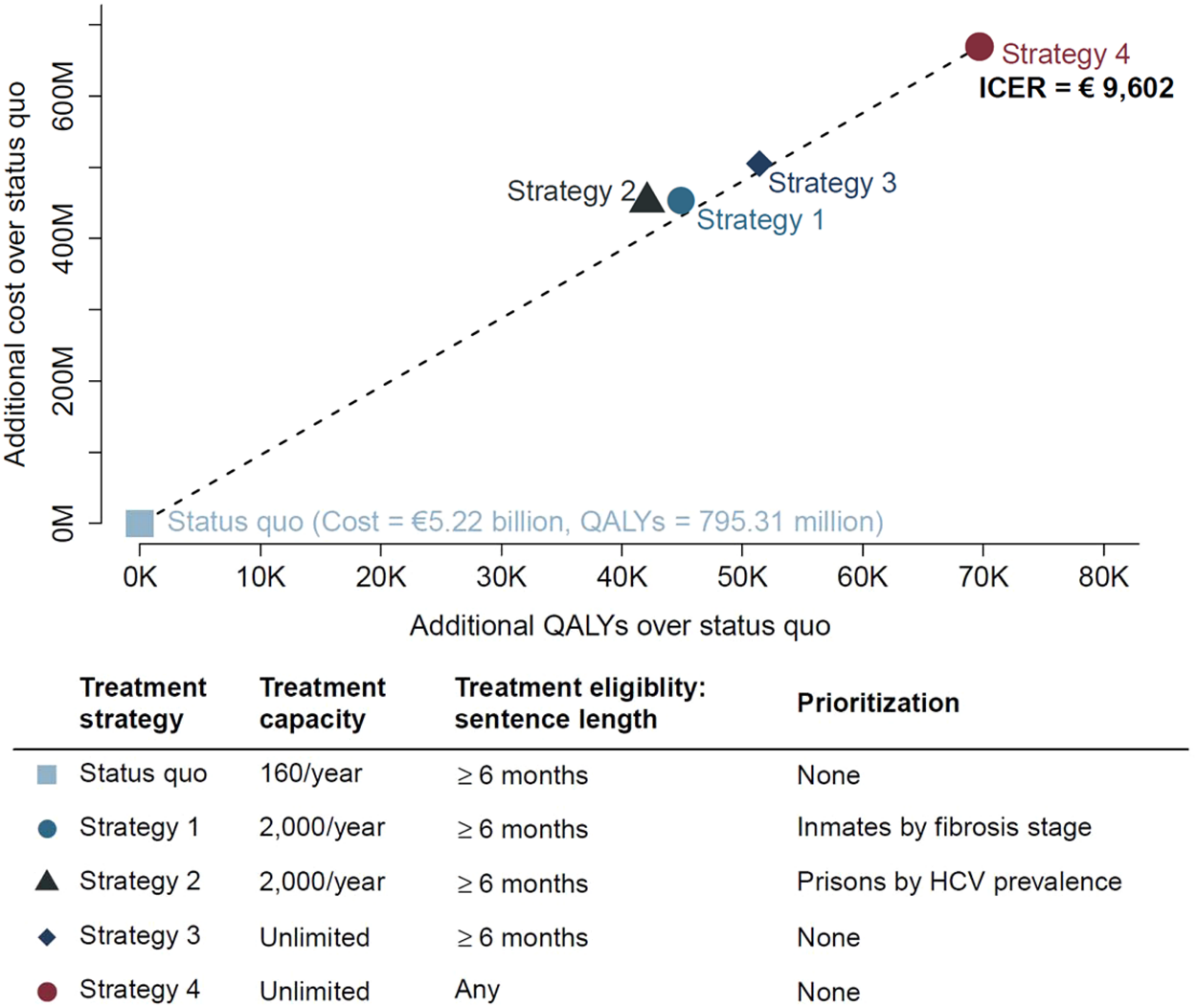
Base case cost-effectiveness analysis results. Abbreviations: QALYs: Quality adjusted life years; ICER: Incremental cost effectiveness ratio.

Compared with status quo, Strategy 1, i.e., scaling-up HCV treatment (2,000/year capacity) to all Spain’s prisons when prioritizing *patients* by their fibrosis stages (F4, F3, F2, F1, F0) and treating those having sentence length of more than 6 months, would increase total population-level QALYs by 44,920, and costs by €451million; Strategy 2, i.e., scaling-up HCV treatment (2,000/year capacity) when prioritizing *prisons* by their HCV prevalence and treating patients having sentence length of more than 6 months, would increase total QALYs by 42,099 and costs by €453 million; Strategy 3, i.e., unlimited treatment capacity, but treating those having sentence length of more than 6 months only, would increase the total QALYs by 51,427 and costs by €505 million; and Strategy 4, i.e., unlimited treatment capacity and treating all irrespective of their sentence length would increase the total QALYs by 69,728 and costs by €670 million, respectively. Strategies 1–3 were dominated by Strategy 4, which yielded an ICER of €9,602 per additional QALY (Fig. 3). Using the commonly-accepted willingness-to-pay threshold range of €21,000–€30,000 in Spain, this strategy was deemed highly cost-effective^16,17^.

### Budget impact analysis

The annual HCV-associated costs (i.e., the cost of HCV screening, treatment and disease management) in Spain’s prisons in 2019 under status quo was €12 million (Table 2). The corresponding cost under strategies to scale-up HCV treatment to all prisons (Strategies 1–4) would be, between €96.2 million and €406.5 million (Table 2). In 2019, the most cost-effective strategy, Strategy 4, would need a budget of €7,900 on screening, €404.6 million on HCV treatment and €1.9 million on HCV disease management. By 2030, the corresponding budget would decrease to €5,800 on screening, €44.7 million on HCV treatment and €0.5 million on HCV disease management.

**Table 2:** Annual HCV–associated cost in Spain’s prisons for years 2019, 2020, 2025, 2030, 2035, 2040, 2045, and 2050 under status quo and different strategies for scaling-up treatment to all prisons.

Cost Type	Strategy	2019	2020	2025	2030	2035	2040	2045	2050
Screening (€)	**Status quo**	7,907	5,876	6,868	2,698	2,150	1,925	1,265	802
	**Strategy 1**	7,907	13,440	13,380	6,024	4,118	2,550	1,929	687
	**Strategy 2**	7,907	9,658	12,295	6,641	4,564	2,782	1,532	689
	**Strategy 3**	7,907	14,412	13,733	6,960	4,478	2,624	1,732	1,205
	**Strategy 4**	7,907	14,695	14,814	5,821	4,302	2,236	1,730	803
Treatment (€)	**Status quo**	8.1 M	7.9 M	7 M	5.9 M	5.1 M	4.3 M	3.7 M	3.1 M
	**Strategy 1**	93.1 M	91.2 M	80 M	40 M	23.3 M	13 M	8.4 M	5.8 M
	**Strategy 2**	93 M	90.5 M	80.3 M	39.6 M	23.4 M	12.8 M	8.3 M	5.8 M
	**Strategy 3**	315.4 M	118.4 M	67.9 M	37.8 M	22.9 M	13 M	8.3 M	5.8 M
	**Strategy 4**	404.6 M	180.2 M	81.7 M	44.7 M	26.2 M	14.7 M	8.9 M	6.1 M
Disease management (€)	**Status quo**	3.7 M	3.5 M	2.8 M	2 M	1.5 M	0.9 M	0.6 M	0.4 M
	**Strategy 1**	3.1 M	2.6 M	1.4 M	0.7 M	0.4 M	0.2 M	0.2 M	0.1 M
	**Strategy 2**	3.2 M	2.7 M	1.4 M	0.7 M	0.4 M	0.2 M	0.2 M	0.1 M
	**Strategy 3**	2.1 M	1.7 M	1.1 M	0.6 M	0.4 M	0.2 M	0.2 M	0.1 M
	**Strategy 4**	1.9 M	1.5 M	0.9 M	0.5 M	0.3 M	0.2 M	0.1 M	0.1 M
Total (€)	**Status quo**	11.8 M	11.5 M	9.8 M	7.9 M	6.6 M	5.2 M	4.3 M	3.5 M
	**Strategy 1**	96.2 M	93.8 M	81.4 M	40.7 M	23.7 M	13.2 M	8.5 M	5.9 M
	**Strategy 2**	96.2 M	93.3 M	81.8 M	40.3 M	23.9 M	13 M	8.5 M	5.9 M
	**Strategy 3**	317.5 M	120.2 M	69.1 M	38.4 M	23.4 M	13.2 M	8.4 M	5.9 M
	**Strategy 4**	406.5 M	181.7 M	82.7 M	45.2 M	26.5 M	14.9 M	9 M	6.2 M

Under status quo, 160 inmates were treated regardless of their fibrosis stages or prisons’ HCV prevalence. Strategy 1 prioritizes inmates by their fibrosis stages (fibrosis scores F4, F3, F2, F1, and F0) with a treatment capacity of 2,000/year, irrespective of the prison or region. Strategy 2 prioritizes prisons by their HCV prevalence with a treatment capacity of 2,000/year, irrespective of fibrosis stages. Strategy 3 considers unlimited capacity. In Strategies 1–3, only those sentenced with more than six months are eligible for treatment. Strategy 4 considers unlimited treatment capacity and assumed everyone, irrespective of their sentence length, is eligible for treatment.

### HCV disease burden

**Figure 4:**
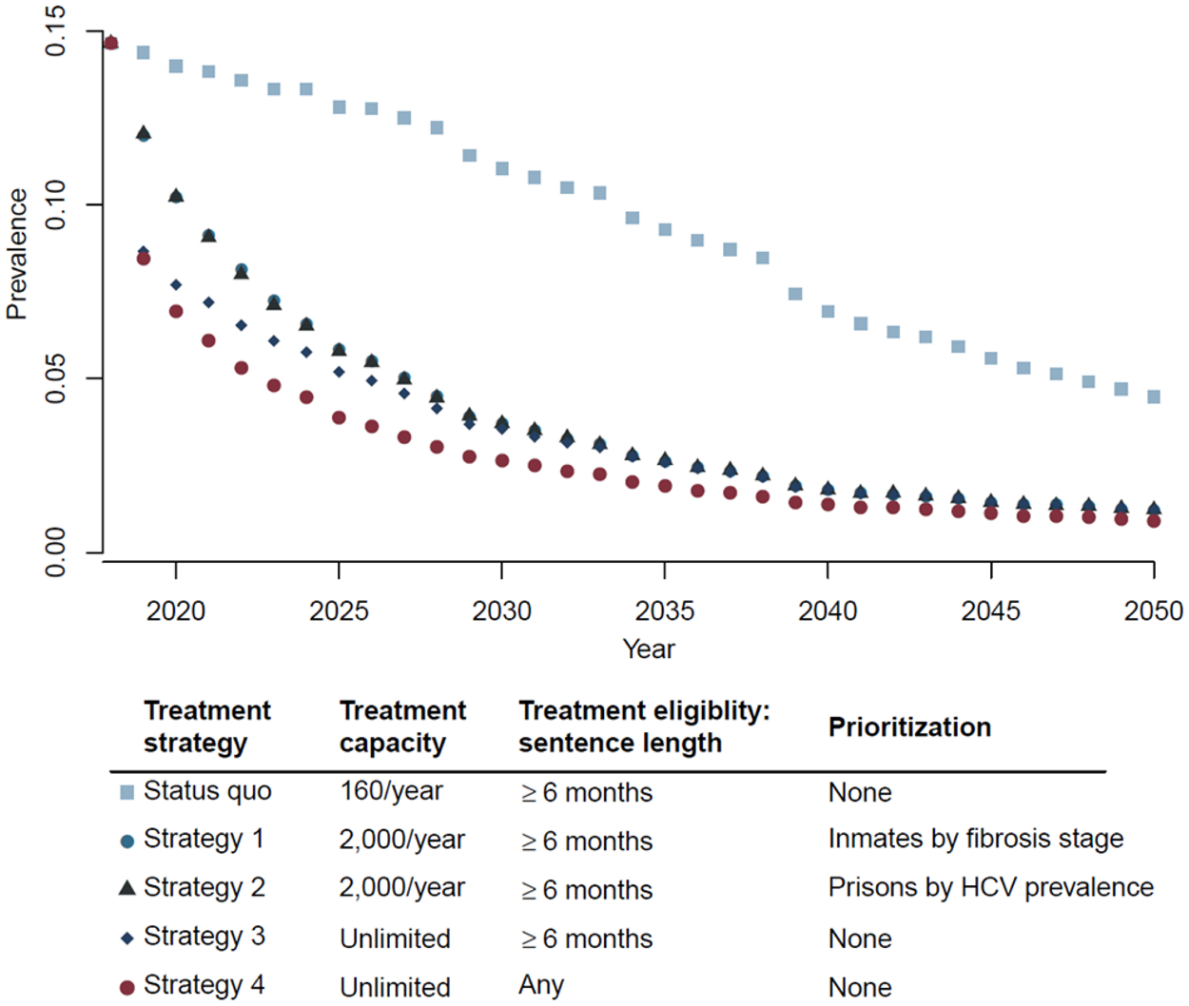
Hepatitis C virus (HCV) prevalence in Spain’s prisons from 2019 to 2050 under status quo and different strategies for scaling-up treatment to all prisons.

Under status quo, HCV prevalence in Spain’s prisons is expected to decrease marginally from 14.4% (i.e., 5,855 viremic people) in 2019 to 11% in 2030 (24% reduction compared with 2019) (Fig. 4). In contrast, scaling-up HCV treatment to all prisons (Strategies 1–4) is predicted to substantially reduce the HCV prevalence in prisons to 2.7–3.7% in 2030 (i.e., 75–82% reduction compared with 2019); Strategy 4 resulted in lowest HCV prevalence. The projected numbers of viremic people in Spain’s prisons in 2030 would be: 4,350 under status quo, 1,460 under Strategy 1, 1,460 under Strategy 2, 1,400 under Strategy 3, and 1,050 under Strategy 4 (Fig. 4).

**Figure 5:**
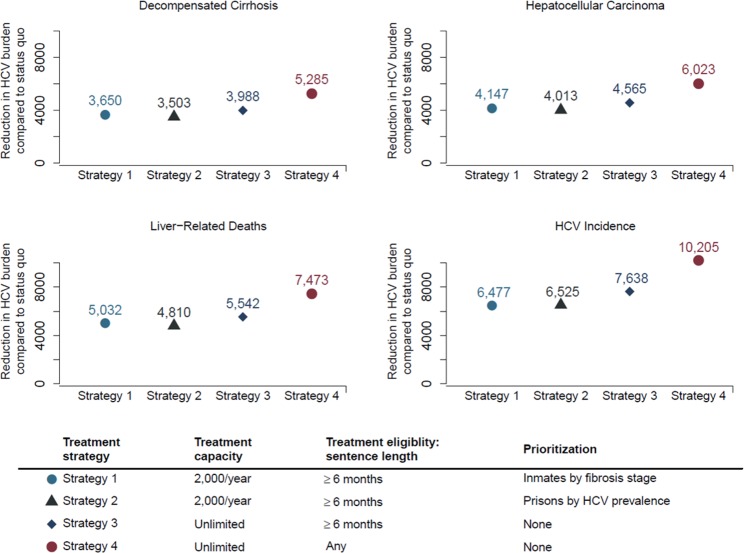
Reduction in hepatitis C disease burden between 2019 and 2050 by scaling-up HCV treatment to all prisons in Spain compared with status quo.

Compared with status quo, scaling-up HCV treatment would reduce decompensated cirrhosis cases by 3,503–5,285 (2.3–3.5% reduction), hepatocellular carcinoma cases by 4,013–6,023 (3–4.5%), liver-related deaths by 5,450–8,281 (3.9–6%), and HCV incidence by 6,477–10,205 (5.6–8.9%) by 2050 in Spain (Fig. 5). We also estimated that among liver-related deaths prevented by scaling-up treatment, 88–90% (4,810–7,473) would have occurred in the community. Similarly, 98–99% (6,371–10,073) of the HCV transmissions averted would have occurred in the community.

### Prison-related HCV transmission and deaths

**Figure 6:**
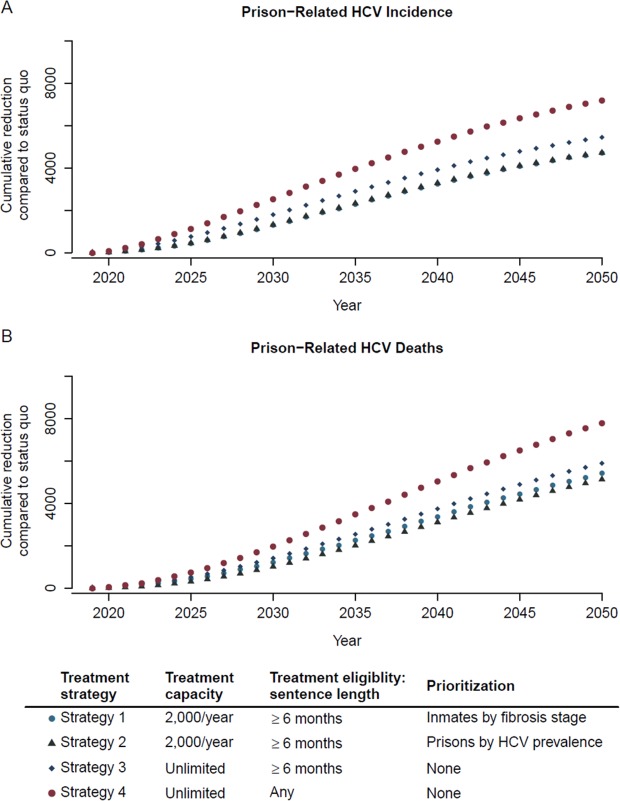
Cumulative reduction in prison-related HCV incidence and prison-related HCV deaths from 2019 to 2050 by scaling-up HCV treatment compared with status quo.

Our model estimated prison-related HCV transmission and deaths; i.e., HCV transmission and deaths because of someone released untreated from prison. Compared with status quo, Strategies 1–4 would respectively reduce prison-related HCV transmission by 4,710 (29%), 4,720 (30%), 5,450 (34%), and 7,180 (45%) between 2019 and 2050 (Fig. 6A). Additionally, compared with status quo, Strategies 1–4 would respectively reduce prison-related HCV deaths by 5,420 (29%), 5,140 (27%), 5,900 (31%), and 7,790 (42%) between 2019 and 2050 (Fig. 6B).

### Sensitivity analysis

We performed one-way sensitivity analysis (Tables 3–7. Among all parameters considered, the cost-effectiveness of the strategies was most sensitive to the quality of life in F0-F3 and decompensated cirrhosis states, HCV transmission probability, and HCV treatment cost. However, for all parameters, the ICER remained below €21,000 per QALY, which implied that the model conclusions were robust against the model parameters. To account for additional different post-treatment programs (including telemedicine, education etc.), we increased the treatment cost by 20% (from €17,126 to €20,551) and found that the ICER of Strategy 4 was €13,585.

We also performed two-way sensitivity analysis on select parameters: HCV transmission probability vs. HCV treatment cost, HCV transmission probability vs. the quality of life of F0-F1 patients, HCV transmission probability vs. the quality of life of F2-F3 patients, HCV transmission probability vs. the quality of life of patients with decompensated cirrhosis, HCV treatment cost vs. the quality of life of F0-F1 patients, HCV treatment cost vs. the quality of life of F2-F3 patients, HCV treatment cost vs. the quality of life of patients with decompensated cirrhosis (Fig. S2). In all cases, Strategy 4 had the highest likelihood of being the most cost-effective strategy.

## Discussion

The recent availability of DAAs offers an unprecedented opportunity to reduce the burden of HCV in Spain, particularly in high-risk populations such as incarcerated people. However, access to treatment remains low in Spain’s prisons. Based on the success of a pilot treatment program, JAILFREE-C^2^, we evaluated the cost-effectiveness of scaling-up HCV treatment in all prisons in Spain. We found that scaling-up HCV treatment with an unlimited treatment capacity and irrespective of sentence length would be highly cost-effective and would reduce HCV prevalence in prisons by 82% and prevent 10,210 new cases of HCV and 8,280 deaths by 2050; the majority of deaths prevented would have occurred in the community. However, this strategy would need the annual budget of €406.5 million in 2019, which would decrease to €45.2 million by 2030.

Earlier studies, primarily from the United States and United Kingdom, have shown that HCV screening and treatment in prison is highly cost-effective and beneficial from societal perspective^4,5,15,18–20^. To our knowledge, our agent-based modeling study provides new information regarding the cost-effectiveness of different strategies to scale-up treatment to all prisons in Spain. We also estimated the benefits of HCV treatment to the society with regards to, reduction in “*prison-related”* HCV transmission and deaths that resulted from individuals whose HCV was not treated while they were incarcerated and went on to infect others in the society after release. We believe our study’s findings are relevant to Spain’s policymakers who are considering expansion of HCV treatment to eliminate HCV.

Our study provides new policy insights. While scaling-up HCV treatment without any capacity will improve outcomes, providing treatment irrespective of inmates’ sentence length — even to those who have less than six months of sentence length — will further reduce HCV transmission, disease burden and mortality society wide. Therefore, it is critical to provide linkage-to-care to persons who start HCV treatment but are released before the treatment completion^21^. Without sufficient linkage, these releasees may not get HCV treatment in the community and could develop advanced sequelae such as decompensated cirrhosis and HCC or transmit HCV to others through different courses such as injection drug use.

Our study has several limitations. First, we limited the horizon of our simulation model to between 2019 and 2050, which could have underestimated the benefits of the treatment strategies evaluated in our study. Second, some parameters were not directly available (e.g., HCV transmission probability), and therefore were estimated through a calibration process. Our sensitivity analysis showed that the conclusions of our study remain robust. Third, we ignored the dynamics of social interactions and assumed age- and IDU-based HCV transmission among the individuals in the community and among inmates. Fourth, because of limited data on the characteristics of those arrested from and released into the community, we used a back-calculation approach that ignored previous incarceration history of people in the community, which is known to be a risk factor for future imprisonment^22^. However, since individual characteristics (age, gender, IDU status and history, and treatment history) in our model did not change over time, we believe our approach will not affect the conclusions of our study.

## Conclusion

Our modeling-based study showed that scaling-up HCV treatment in all prisons in Spain is highly cost-effective. Unrestricted treatment to all HCV-infected persons in prisons regardless of their sentence length will have the maximum impact of reducing the burden of HCV in the whole society, resulting in reduction of HCV prevalence in prisons by more than 80% and averting 8,300 HCV deaths and 10,200 HCV transmission by 2050.

## Supplementary information

Improved Health Outcomes from Hepatitis C Treatment Scale-Up in Spain’s Prisons: A Cost-Effectiveness Study

## Data Availability

All the data used in this study are available in published literature and reports.
