# Supplementary material for: Improved Health Outcomes from Hepatitis C Treatment Scale-Up in Spain’s Prisons: A Cost-Effectiveness Study
Source: Sci Rep. 2019 Nov 14;9:16849. doi: 10.1038/s41598-019-52564-0 (PMC6856347; doi:10.1038/s41598-019-52564-0)
Supplement: Supplementary file 1 — Improved Health Outcomes from Hepatitis C Treatment Scale-Up in Spain’s Prisons: A Cost-Effectiveness Study [file 41598_2019_52564_MOESM1_ESM.docx]

**Improved Health Outcomes from Hepatitis C Treatment Scale-Up in Spain’s Prisons:
A Cost-Effectiveness Study**

Ozden O. Dalgic,^1^ Sumeyye Samur,^1^ Anne C. Spaulding,^3^ Susana Llerena,^4^ Carmen Cobo,^5^ Turgay Ayer,^6^ Mark S. Roberts,^7^ Javier Crespo,^4^* Jagpreet Chhatwal^1^*

^1^Institute for Technology Assessment, Massachusetts General Hospital, Harvard Medical School, Boston, Massachusetts, USA

^3^Department of Epidemiology, Rollins School of Public Health, Emory University, Atlanta, Georgia, USA.

^4^Department of Gastroenterology and Hepatology, Marques de Valdecilla University Hospital, Santander, Spain

^5^Medical Service, El Dueso Penitentiary Centre, Santoña, Spain.

^6^H. Milton Stewart School of Industrial and Systems Engineering, Georgia Institute of Technology, Atlanta, Georgia, USA.

^7^Department of Health Policy and Management, University of Pittsburgh, Pittsburgh, Pennsylvania, USA.

*co-senior authors

**Supplementary Section 1. Model Details**

We adapted a previously developed agent-based simulation model – treatment as prevention of HCV (TapHCV), to simulate the landscape of hepatitis C virus (HCV) in both the prisons and the general population in Spain.^1^ Agents in our model represented individuals in prisons or community, with or without HCV. The model was developed in C++, a general-purpose computer programming language,^2,3^ and consisted of the following major components: HCV screening, HCV disease transmission and progression, HCV treatment, and simulation of prison and general population dynamics.

**Baseline Population**

We simulated 8 million agents in the community and 7,048 agents for the prisons to define our population and scaled-up all results by a factor of 5.8 to project national-level outcomes. We stratified our model’s population by age, gender, fibrosis stages, prevalence of HCV, range of HCV genotype, treatment acceptability, treatment experiences, injection drug use, and incarceration history. We also considered population growth in the society. Newborns were added each year based on the annual birth rates in the Spain.^4^ For people who inject drug (PWIDs) and incarcerated individuals, we adjusted the baseline mortality rates by standardized mortality ratio (SMR).^5,6^

We defined the baseline distribution of the four most common HCV genotypes (G1, G2, G3 and G4), and chronic HCV stages using METAVIR fibrosis scores (no fibrosis [F0], portal fibrosis without septa [F1], portal fibrosis with few septa [F2], numerous septa without fibrosis [F3], or compensated cirrhosis [F4]), advanced HCV states (decompensated cirrhosis, hepatocellular carcinoma, liver transplant, and liver-related death) and treatment history (previously treated or treatment-naïve) using published studies.^7-15^

**Prison system and HCV prevalence in prisons in Spain**

In Spain, there are 17 autonomous communities, each having multiple correctional facilities. The proportion of the HCV infected population in the prisons varies between 2.2-25.7% (**Table S1**).

In each community, some functions are transferred from the central government to autonomous (community) governments. In two communities, namely Euskadi and Catalonia, prison health functions are transferred to the autonomous governments and funding of the prison depends on the Ministry of Health. In the rest of the communities of Spain, prison health functions are not transferred, it depends on the interior ministry and is paid by the Central government.

**Table S1** Regions and HCV prevalence and of the correctional facilities in Spain^16^

| **Facility** | **Region** | **HCV Prevalence** |
| --- | --- | --- |
| Albolote | Andalusia | 17.9% |
| Alcalá guad. | Andalusia | 13.3% |
| Algeciras | Andalusia | 12.4% |
| Córdoba | Andalusia | 13.0% |
| Huelva | Andalusia | 17.0% |
| Jaén | Andalusia | 15.2% |
| Puerto-2 | Andalusia | 15.3% |
| Seville | Andalusia | 12.1% |
| Seville Moron | Andalusia | 22.5% |
| Daroca | Aragon | 24.3% |
| Teruel | Aragon | 8.9% |
| Zaragoza | Aragon | 12.5% |
| Villabona | Asturias | 21.6% |
| Ibiza | Balearic Islands | 5.6% |
| Palma Mall. | Balearic Islands | 21.7% |
| Bilbao | Basque country | 9.5% |
| San Sebastián | Basque country | 10.7% |
| Las Palmas 2 | Canary Islands | 12.0% |
| Tenerife | Canary Islands | 17.9% |
| El Dueso | Cantabria | 18.0% |
| Alcazar | Castile-La Mancha | 10.6% |
| Cuenca | Castile-La Mancha | 18.2% |
| Herrera | Castile-La Mancha | 15.6% |
| Ocaña-1 | Castile-La Mancha | 12.3% |
| Ocaña-2 | Castile-La Mancha | 22.8% |
| Ávila | Castile-Leon | 16.8% |
| Burgos | Castile-Leon | 16.0% |
| León | Castile-Leon | 16.7% |
| Segovia | Castile-Leon | 15.1% |
| Soria | Castile-Leon | 25.7% |
| Valladolid | Castile-Leon | 10.4% |
| Badajoz | Extremadura | 14.2% |
| Cáceres | Extremadura | 15.0% |
| A Lama | Galicia | 19.3% |
| Lugo Bonxe | Galicia | 18.1% |
| Lugo Mont. | Galicia | 15.7% |
| Orense | Galicia | 16.2% |
| Teixeiro | Galicia | 24.7% |
| Madrid 1 | Madrid | 5.6% |
| Madrid 2 | Madrid | 7.7% |
| Madrid 4 | Madrid | 16.7% |
| Madrid 5 | Madrid | 8.6% |
| Madrid 6 | Madrid | 16.2% |
| Madrid 7 | Madrid | 12.3% |
| Melilla | Melilla (Andalusia) | 2.2% |
| Murcia 1 | Murcia | 17.2% |
| Murcia 2 | Murcia | 13.1% |
| Pamplona | Navarra | 10.3% |
| Alicante-2 | Valencia | 14.9% |
| Castellón 2 | Valencia | 17.1% |
| Valencia | Valencia | 11.5% |

**HCV Transmission and Disease Progression**

In the TapHCV mode, we simulated HCV transmission separately in prisons and in the general population. For that purpose, we probabilistically formed pairs between individuals. HCV-infected individual inside prisons could only pair with those inside prisons, and vice versa. PWIDs had a higher probability of pairing with other PWIDs, and vice versa. At each month, an HCV-infected individual contact with one individual from the susceptible pool. The susceptible pool consists of individuals who were never infected with HCV as well as those who were previously infected but treated. Previously-treated individuals have a higher chance to be paired. At each month, during a contact between HCV-infected and susceptible individual, the virus could be transmitted with a certain probability, $P_{trans}$. The transmission probability was dependent on the HCV-infected individual’s awareness status, the susceptible individual’s prior treatments status, and the injection drug use statuses of the both individuals.

$P_{trans}$ is calculated as follows:

$$P_{trans}=P_{D}\times\left( 1-A_{I} \right)\times\left( 1-T_{E} \right).$$

Here, $A_{I}$ is the reduction factor attributed to the awareness status of HCV-infected individual, which is equal to 0 if the individual is unaware. Similarly, $T_{E}$ is the reduction factor for susceptible individuals with prior treatment status, which is equal to zero if the susceptible individual was not treated before. Finally, $P_{D}$ is the probability of the transmission and calculated as follows:

$$P_{D}=1-\left( 1-P_{0} \right)^{D_{I}\times D_{E}}$$

where $D_{I}$ and $D_{E}$ were the hazard ratio for injection drug use of an HCV-infected and a susceptible individual, respectively. $P_{0}$ denotes the baseline transmission probability.

All newly-infected individuals started with the acute phase of HCV. The acute infection lasted for six months and ended with either a recovery at 25% chance or otherwise advancing to the chronic phase of HCV disease.^17^ The chronic disease progressed through different stages of fibrosis, as defined by METAVIR scale units, F0 to F4. Patients at METAVIR fibrosis score F3 and F4 could develop advanced diseases such as decompensated cirrhosis, hepatocellular carcinoma.^17-28^ Patients with decompensated cirrhosis or hepatocellular carcinoma were eligible for receiving a liver transplant or they could die because of high liver-related mortality.^29-33^

Disease progressed at the same rate for patients who failed to achieve SVR as in untreated patients. Those who achieved SVR were assumed to transition into normal health status if they were not cirrhotic. In cirrhotic patients, we assumed that the disease would progress even after achieving SVR, though at a slower rate.^17^

**Arrest and Release Dynamics**

The probability of incarceration, i.e., the probability of someone getting arrested from the community, was back-calculated such that average age of inmates and gender distribution, prevalence of PWIDs and former PWIDs in prisons, and prevalence of HCV antibody in prisons remained stable over time. For this purpose, we first defined all possible inmate profiles (e.g., Profile 1: [age: 30, gender: male, IDU status: active PWID, IDU history: yes, treatment history: none] or Profile 2: [age: 30, gender: male, IDU status: active PWID, IDU history: yes, treatment history: none], etc.). We then estimated the likelihood of each profile being observed in the prisons, which was defined as the proportion of inmates with that profile in a given prison. The probability of incarceration of someone (from community) having a specific profile is defined as the likelihood of that profile in the prison divided by the number of those profiles in the community. The calculations of the incarceration probability ($\boldsymbol{P}_{\boldsymbol{t}}^{\boldsymbol{i}}$) is presented as follows.

| $\boldsymbol{i}$**:** | Profile, any combination of age, gender, IDU status, IDU history, and treatment history of patients. Age is considered as age-groups (i.e., 0-4, 5-9, 10-15, …) |
| --- | --- |
| $\boldsymbol{N}_{\boldsymbol{t}}^{\boldsymbol{i}}$**:** | Number of people with profile $i$ in the community at time $t$. |
| $\boldsymbol{S}^{\boldsymbol{i}}$**:** | Probability of profile $i$ in the sampled prison population  $\frac{Number of inmates with profile i in the sample populaton}{Number of inmates in the sampled size}$ |
| $\boldsymbol{P}_{\boldsymbol{t}}^{\boldsymbol{i}}$**:** | Probability of incarceration of profile $i$ at time $t$. |

$$\boldsymbol{P}_{\boldsymbol{t}}^{\boldsymbol{i}}\boldsymbol{=}\frac{\boldsymbol{S}_{\boldsymbol{p}}}{N_{t}^{i}}$$

Arrested individuals were released after their sentence was complete, which is defined in Table S2.

**Table S2** Length of sentence in Spain’s prisons

| Length of Sentence^6^ | Distribution |
| --- | --- |
| 1 to 6 months | 58.23% |
| 6 months to 2 years | 34.08% |
| 2 to 4 years | 6.27% |
| 4 to 10 years | 1.24% |
| 10 to 20 years | 0.17% |
| 20 to 25 years | 0.01% |

**Details of JAILFREE-C project**

The JAILFREE-C project is focused on the El Dueso Prison. A total of 847 inmates agreed to participate and 99.5% were tested for HCV, and 86 (10.2%) patients were detected with active infection. Treatment was started in 69 patients (the rest were not initiated because their prison stays was less than 30 days). The SVR was achieved in 64 patients (3 of them needed rescue with other combinations of antivirals) and 2 patients were lost follow-up.

**Model Validation**

Originally, several transmission-related parameters were already calibrated to mimic the HCV dynamics in the prisons and community in the U.S.^1^ However, to estimate the HCV prevalence in the Spain prisons, we recalibrated the model by changing the annual number of people receiving HCV treatment in prisons under status quo such that the model-predicted HCV prevalence in prisons matched with the HCV prevalence reported in the Ministry Interior reports for years 2010 to 2015. We found that 160 individuals received HCV treatment each year from 2010 to 2015 (**Figure S1**).


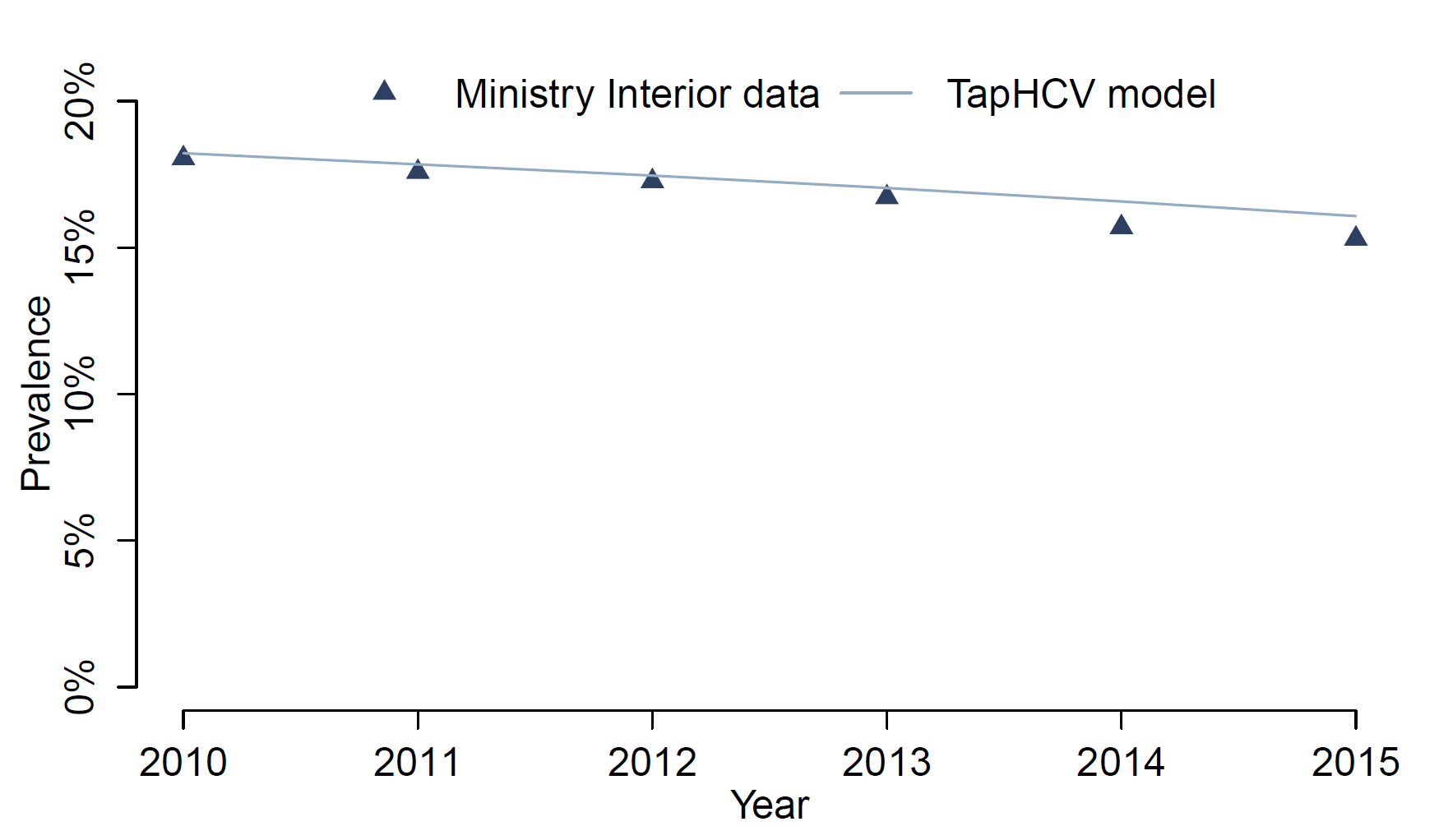


**Figure S1**. Model validation with the published data. We compared our model-predicted results with that of reported values from Ministry Interior. Our model-predicted results matched closely with the reported values.

**Section 2 Sensitivity Analysis Results**

**Table S3.** Results of one-way sensitivity analysis showing decompensated cirrhosis cases averted.

| **Parameter** | **Value  (Low/ High)** | **Decompensated Cirrhosis Cases** | **Decompensated Cirrhosis Cases Averted** | | | |
| --- | --- | --- | --- | --- | --- | --- |
|  |  |  | **(Compared with Status Quo)** | | | |
|  |  | **Status Quo** | **Strategy 1** | **Strategy 2** | **Strategy 3** | **Strategy 4** |
| **Base Case** |  | **48 951** | **3 650** | **3 503** | **3 988** | **5 285** |
| **HCV-Associated Agent Characteristics** | |  |  |  |  |  |
| Awareness: Community | 0.05 0.55 | 60 188 43 931 | 4 064 3 344 | 3 946 3 203 | 4 628 3 631 | 6 161 4 749 |
| Awareness: Prisons | 0.7 0.9 | 49 103 48 998 | 3 646 3 646 | 3 521 3 482 | 3 984 3 990 | 5 282 5 237 |
| Treatment experience: Inmates | 0.131 0.331 | 48 778 48 921 | 3 667 3 702 | 3 519 3 521 | 3 998 4 016 | 5 271 5 294 |
| HCV transmission probability | 0.000085 0.000425 | 42 405 60 170 | 2 243 5 554 | 2 113 5 447 | 2 430 6 277 | 3 253 8 581 |
| Self-clearance probability | 0.23 0.28 | 49 290 48 420 | 3 741 3 456 | 3 600 3 379 | 4 110 3 824 | 5 357 5 060 |
| Newborn infection rate | 0.0061% 0.018% | 48 926 49 011 | 3 660 3 664 | 3 495 3 512 | 3 991 4 036 | 5 245 5 262 |
| **Transition Probabilities** |  |  |  |  |  |  |
| F3 to hepatocellular carcinoma (annual) | 0.003 0.014 | 49 967 47 768 | 3 707 3 604 | 3 567 3 422 | 4 074 3 921 | 5 382 5 152 |
| Compensated cirrhosis (F4) to decompensated cirrhosis (annual) | 0.01 0.079 | 28 881 69 764 | 1 916 5 929 | 1 836 5 700 | 2 141 6 508 | 2 772 8 456 |
| Compensated cirrhosis (F4) to hepatocellular carcinoma (annual) | 0.01 0.079 | 61 458 28 200 | 4 416 2 338 | 4 187 2 247 | 4 844 2 519 | 6 362 3 273 |
| SVR after cirrhosis to decompensated cirrhosis (annual) | 0.002 0.036 | 48 951 60 925 | 3 650 2 430 | 3 503 2 382 | 3 988 2 723 | 5 285 3 520 |
| SVR after cirrhosis to hepatocellular carcinoma (annual) | 0.002 0.013 | 48 983 48 844 | 3 645 3 679 | 3 497 3 527 | 4 009 4 047 | 5 261 5 285 |
| Decompensated cirrhosis to hepatocellular carcinoma (annual) | 0.03 0.083 | 48 969 48 892 | 3 629 3 652 | 3 474 3 478 | 3 975 3 995 | 5 251 5 249 |
| Decompensated cirrhosis to liver transplant (annual) | 0.01 0.062 | 48 928 48 984 | 3 646 3 698 | 3 531 3 532 | 3 969 4 038 | 5 257 5 324 |
| Decompensated cirrhosis (first year) to liver-related death (annual) | 0.065 0.19 | 48 968 48 893 | 3 666 3 614 | 3 526 3 460 | 3 997 3 961 | 5 290 5 213 |
| Decompensated cirrhosis (subsequent year) to liver-related death (annual) | 0.065 0.19 | 48 988 48 838 | 3 663 3 630 | 3 461 3 533 | 3 985 3 988 | 5 264 5 241 |
| Hepatocellular carcinoma to liver transplant (annual) | 0 0.14 | 48 909 49 010 | 3 692 3 652 | 3 529 3 503 | 4 026 4 007 | 5 232 5 250 |
| Hepatocellular carcinoma to liver-related death (annual) | 0.33 0.86 | 48 996 48 843 | 3 662 3 660 | 3 524 3 532 | 4 033 4 005 | 5 258 5 288 |
| Liver transplant (first year) to liver-related death (annual) | 0.06 0.42 | 48 933 48 915 | 3 645 3 642 | 3 476 3 465 | 4 005 3 999 | 5 218 5 241 |
| Liver transplant (subsequent year) to liver-related death (annual) | 0.024 0.11 | 48 960 48 951 | 3 660 3 690 | 3 507 3 520 | 4 023 4 040 | 5 281 5 277 |
| **Agent's Behavior** |  |  |  |  |  |  |
| F0 HCV diagnosis probability | 0.02787 0.04606 | 48 344 47 995 | 3 568 3 517 | 3 429 3 365 | 3 880 3 854 | 5 140 5 064 |
| F1 HCV diagnosis probability | 0.02236 0.03702 | 48 145 47 657 | 3 579 3 489 | 3 411 3 350 | 3 892 3 839 | 5 138 5 017 |
| F2 HCV diagnosis probability | 0.03179 0.05246 | 47 860 47 194 | 3 655 3 527 | 3 473 3 402 | 3 953 3 856 | 5 188 5 101 |
| F3 HCV diagnosis probability | 0.03471 0.05724 | 47 152 46 155 | 3 585 3 542 | 3 415 3 405 | 3 916 3 854 | 5 164 5 133 |
| F4 HCV diagnosis probability | 0.12439 0.19926 | 41 554 39 835 | 3 476 3 382 | 3 329 3 247 | 3 781 3 695 | 4 987 4 874 |
| Aware reduction factor | 0.25 0.75 | 50 628 47 362 | 4 104 3 274 | 3 965 3 095 | 4 530 3 547 | 5 967 4 611 |
| Treatment reduction factor | 0 1 | 48 951 47 108 | 3 650 3 388 | 3 503 3 242 | 3 988 3 656 | 5 285 4 808 |

*Decompensated cirrhosis cases averted in comparison with status quo were represented in the sensitivity analysis. Note that number of decompensated cirrhosis cases averted under the base case may not necessarily reside in the range obtained by low and high parameter values since the overall number of decompensated cirrhosis cases under status quo also changed for each parameter in the sensitivity analysis. Therefore, trends for low and high values should be evaluated independently. Lastly, results could be influenced by first-order uncertainty that could result in inconsistent trends.

Under status quo, 160 inmates were treated regardless of their fibrosis stages or prisons’ HCV prevalence. Strategy 1 prioritizes inmates by their fibrosis stages (fibrosis scores F4, F3, F2, F1, and F0) with a treatment capacity of 2,000/year, irrespective of the prison or region. Strategy 2 prioritizes prisons by their HCV prevalence with a treatment capacity of 2,000/year, irrespective of fibrosis stages. Strategy 3 considers unlimited capacity. In Strategies 1–3, only those sentenced with more than six months are eligible for treatment. Strategy 4 considers unlimited treatment capacity and assumed everyone, irrespective of their sentence length, is eligible for treatment.

Abbreviations: HCV, hepatitis C virus; METAVIR, meta-analysis of histologic data in viral hepatitis; F0-F4, METAVIR fibrosis score; SVR, sustained virologic response.

**Table S4.** Results of one-way sensitivity analysis showing hepatocellular carcinoma cases averted.

| **Parameter** | **Value  (Low/ High)** | **Hepatocellular Carcinoma**  **Cases** | **Hepatocellular Carcinoma Cases*** | | | |
| --- | --- | --- | --- | --- | --- | --- |
|  |  |  | **(Compared with Status Quo)** | | | |
|  |  | **Status Quo** | **Strategy 1** | **Strategy 2** | **Strategy 3** | **Strategy 4** |
| **Base Case** |  | **73 443** | **4 147** | **4 013** | **4 565** | **6 023** |
| **HCV-Associated Agent Characteristics** | |  |  |  |  |  |
| Awareness: Community | 0.05 0.55 | 88 211 66 975 | 4 616 3 831 | 4 418 3 691 | 5 227 4 213 | 7 004 5 489 |
| Awareness: Prisons | 0.7 0.9 | 73 667 73 522 | 4 093 4 143 | 3 994 4 005 | 4 523 4 533 | 6 009 6 017 |
| Treatment experience: Inmates | 0.131 0.331 | 73 437 73 503 | 4 130 4 116 | 4 025 3 942 | 4 583 4 545 | 6 058 5 980 |
| HCV transmission probability | 0.000085 0.000425 | 65 010 87 798 | 2 547 6 287 | 2 401 6 102 | 2 796 7 105 | 3 713 9 743 |
| Self-clearance probability | 0.23 0.28 | 73 927 72 762 | 4 253 3 979 | 4 102 3 827 | 4 699 4 401 | 6 157 5 787 |
| Newborn infection rate | 0.0061% 0.018% | 73 430 73 569 | 4 168 4 182 | 3 953 4 005 | 4 595 4 622 | 6 005 6 034 |
| **Transition Probabilities** |  |  |  |  |  |  |
| F3 to hepatocellular carcinoma (annual) | 0.003 0.014 | 69 248 77 782 | 3 911 4 465 | 3 748 4 296 | 4 314 4 908 | 5 653 6 463 |
| Compensated cirrhosis (F4) to decompensated cirrhosis (annual) | 0.01 0.079 | 77 859 64 704 | 4 343 3 795 | 4 180 3 637 | 4 822 4 167 | 6 320 5 396 |
| Compensated cirrhosis (F4) to hepatocellular carcinoma (annual) | 0.01 0.079 | 54 149 93 714 | 2 617 6 383 | 2 502 6 186 | 2 876 7 016 | 3 816 9 118 |
| SVR after cirrhosis to decompensated cirrhosis (annual) | 0.002 0.036 | 73 443 76 203 | 4 147 3 885 | 4 013 3 768 | 4 565 4 343 | 6 023 5 720 |
| SVR after cirrhosis to hepatocellular carcinoma (annual) | 0.002 0.013 | 71 704 77 515 | 4 321 3 730 | 4 138 3 649 | 4 724 4 163 | 6 286 5 438 |
| Decompensated cirrhosis to hepatocellular carcinoma (annual) | 0.03 0.083 | 64 811 76 012 | 3 723 4 271 | 3 560 4 114 | 4 123 4 747 | 5 374 6 232 |
| Decompensated cirrhosis to liver transplant (annual) | 0.01 0.062 | 74 547 70 771 | 4 179 4 006 | 4 021 3 864 | 4 616 4 456 | 6 059 5 874 |
| Decompensated cirrhosis (first year) to liver-related death (annual) | 0.065 0.19 | 76 172 73 221 | 4 288 4 122 | 4 119 3 934 | 4 732 4 542 | 6 230 5 957 |
| Decompensated cirrhosis (subsequent year) to liver-related death (annual) | 0.065 0.19 | 78 371 68 646 | 4 266 3 942 | 4 120 3 801 | 4 711 4 369 | 6 248 5 796 |
| Hepatocellular carcinoma to liver transplant (annual) | 0 0.14 | 73 438 73 493 | 4 186 4 107 | 4 032 3 944 | 4 590 4 554 | 6 029 5 984 |
| Hepatocellular carcinoma to liver-related death (annual) | 0.33 0.86 | 73 523 73 337 | 4 170 4 198 | 4 021 4 009 | 4 542 4 589 | 5 994 6 042 |
| Liver transplant (first year) to liver-related death (annual) | 0.06 0.42 | 73 484 73 420 | 4 207 4 145 | 4 035 3 978 | 4 626 4 521 | 6 034 5 968 |
| Liver transplant (subsequent year) to liver-related death (annual) | 0.024 0.11 | 73 463 73 435 | 4 113 4 134 | 3 962 3 962 | 4 569 4 583 | 5 998 5 998 |
| **Agent's Behavior** |  |  |  |  |  |  |
| F0 HCV diagnosis probability | 0.02787 0.04606 | 72 678 72 264 | 4 055 4 028 | 3 901 3 872 | 4 441 4 437 | 5 869 5 831 |
| F1 HCV diagnosis probability | 0.02236 0.03702 | 72 364 71 724 | 4 082 3 988 | 3 900 3 833 | 4 459 4 384 | 5 896 5 772 |
| F2 HCV diagnosis probability | 0.03179 0.05246 | 71 846 70 917 | 4 089 4 021 | 3 928 3 891 | 4 490 4 458 | 5 924 5 871 |
| F3 HCV diagnosis probability | 0.03471 0.05724 | 70 750 69 332 | 4 115 4 020 | 3 948 3 897 | 4 531 4 448 | 5 934 5 862 |
| F4 HCV diagnosis probability | 0.12439 0.19926 | 66 016 64 240 | 3 992 3 919 | 3 814 3 754 | 4 377 4 302 | 5 719 5 638 |
| Aware reduction factor | 0.25 0.75 | 75 590 71 372 | 4 606 3 657 | 4 481 3 480 | 5 141 4 006 | 6 754 5 266 |
| Treatment reduction factor | 0 1 | 73 443 71 281 | 4 147 3 876 | 4 013 3 733 | 4 565 4 210 | 6 023 5 558 |

*Hepatocellular carcinoma cases averted in comparison with status quo were represented in the sensitivity analysis. Note that number of hepatocellular carcinoma case under the base case may not necessarily reside in the range obtained by low and high parameter values since the overall number of hepatocellular cases under status quo also changed for each parameter in the sensitivity analysis. Therefore, trends for low and high values should be evaluated independently. Lastly, results could be influenced by first-order uncertainty that could result in inconsistent trends.

Under status quo, 160 inmates were treated regardless of their fibrosis stages or prisons’ HCV prevalence. Strategy 1 prioritizes inmates by their fibrosis stages (fibrosis scores F4, F3, F2, F1, and F0) with a treatment capacity of 2,000/year, irrespective of the prison or region. Strategy 2 prioritizes prisons by their HCV prevalence with a treatment capacity of 2,000/year, irrespective of fibrosis stages. Strategy 3 considers unlimited capacity. In Strategies 1–3, only those sentenced with more than six months are eligible for treatment. Strategy 4 considers unlimited treatment capacity and assumed everyone, irrespective of their sentence length, is eligible for treatment.

Abbreviations: HCV, hepatitis C virus; METAVIR, meta-analysis of histologic data in viral hepatitis; F0-F4, METAVIR fibrosis score; SVR, sustained virologic response.

**Table S5.** Results of one-way sensitivity analysis showing liver-related deaths averted.

| **Parameter** | **Value  (Low/ High)** | **Liver-Related Deaths** | **Liver-Related Deaths Averted*** | | | |
| --- | --- | --- | --- | --- | --- | --- |
|  |  |  | **(Compared with Status Quo)** | | | |
|  |  | **Status Quo** | **Strategy 1** | **Strategy 2** | **Strategy 3** | **Strategy 4** |
| **Base Case** |  | **114 135** | **5 032** | **4 810** | **5 542** | **7 473** |
| **HCV-Associated Agent Characteristics** | |  |  |  |  |  |
| Awareness: Community | 0.05 0.55 | 135 431 104 889 | 5 613 4 648 | 5 347 4 426 | 6 366 5 075 | 8 704 6 789 |
| Awareness: Prisons | 0.7 0.9 | 114 478 114 259 | 5 000 5 012 | 4 806 4 791 | 5 542 5 516 | 7 474 7 444 |
| Treatment experience: Inmates | 0.131 0.331 | 114 122 114 184 | 5 053 5 026 | 4 845 4 760 | 5 571 5 532 | 7 513 7 443 |
| HCV transmission probability | 0.000085 0.000425 | 103 205 132 118 | 3 236 7 301 | 3 023 7 067 | 3 535 8 274 | 4 809 11 603 |
| Self-clearance probability | 0.23 0.28 | 114 764 113 274 | 5 155 4 810 | 4 930 4 626 | 5 704 5 341 | 7 618 7 187 |
| Newborn infection rate | 0.0061% 0.018% | 114 134 114 302 | 5 057 5 061 | 4 769 4 810 | 5 562 5 606 | 7 451 7 477 |
| **Transition Probabilities** |  |  |  |  |  |  |
| F3 to hepatocellular carcinoma (annual) | 0.003 0.014 | 110 308 118 105 | 4 850 5 241 | 4 610 5 004 | 5 389 5 776 | 7 207 7 771 |
| Compensated cirrhosis (F4) to decompensated cirrhosis (annual) | 0.01 0.079 | 101 437 127 810 | 4 335 5 840 | 4 166 5 615 | 4 825 6 442 | 6 460 8 560 |
| Compensated cirrhosis (F4) to hepatocellular carcinoma (annual) | 0.01 0.079 | 101 552 124 549 | 4 119 6 269 | 3 896 6 017 | 4 541 6 875 | 6 141 9 122 |
| SVR after cirrhosis to decompensated cirrhosis (annual) | 0.002 0.036 | 114 135 124 395 | 5 032 4 132 | 4 810 4 002 | 5 542 4 629 | 7 473 6 188 |
| SVR after cirrhosis to hepatocellular carcinoma (annual) | 0.002 0.013 | 112 546 117 887 | 5 137 4 676 | 4 885 4 489 | 5 670 5 205 | 7 680 6 965 |
| Decompensated cirrhosis to hepatocellular carcinoma (annual) | 0.03 0.083 | 113 711 114 161 | 4 952 5 025 | 4 708 4 795 | 5 471 5 583 | 7 334 7 479 |
| Decompensated cirrhosis to liver transplant (annual) | 0.01 0.062 | 114 956 112 220 | 5 048 4 920 | 4 852 4 689 | 5 580 5 466 | 7 474 7 353 |
| Decompensated cirrhosis (first year) to liver-related death (annual) | 0.065 0.19 | 114 778 114 043 | 4 955 4 971 | 4 726 4 734 | 5 464 5 502 | 7 384 7 397 |
| Decompensated cirrhosis (subsequent year) to liver-related death (annual) | 0.065 0.19 | 113 326 113 923 | 4 825 5 184 | 4 590 4 947 | 5 325 5 739 | 7 197 7 739 |
| Hepatocellular carcinoma to liver transplant (annual) | 0 0.14 | 115 377 111 783 | 5 165 4 779 | 4 940 4 536 | 5 671 5 302 | 7 632 7 118 |
| Hepatocellular carcinoma to liver-related death (annual) | 0.33 0.86 | 114 375 113 224 | 4 911 5 295 | 4 690 5 061 | 5 383 5 833 | 7 244 7 838 |
| Liver transplant (first year) to liver-related death (annual) | 0.06 0.42 | 114 037 114 915 | 5 050 5 134 | 4 792 4 874 | 5 558 5 630 | 7 433 7 573 |
| Liver transplant (subsequent year) to liver-related death (annual) | 0.024 0.11 | 112 229 116 660 | 4 961 5 107 | 4 715 4 836 | 5 484 5 660 | 7 382 7 610 |
| **Agent's Behavior** |  |  |  |  |  |  |
| F0 HCV diagnosis probability | 0.02787 0.04606 | 113 205 112 670 | 4 935 4 881 | 4 687 4 625 | 5 418 5 384 | 7 300 7 214 |
| F1 HCV diagnosis probability | 0.02236 0.03702 | 112 846 112 017 | 4 985 4 846 | 4 734 4 607 | 5 454 5 360 | 7 358 7 191 |
| F2 HCV diagnosis probability | 0.03179 0.05246 | 112 160 110 977 | 4 952 4 865 | 4 717 4 648 | 5 454 5 391 | 7 358 7 271 |
| F3 HCV diagnosis probability | 0.03471 0.05724 | 110 778 108 926 | 4 976 4 874 | 4 751 4 687 | 5 489 5 396 | 7 375 7 299 |
| F4 HCV diagnosis probability | 0.12439 0.19926 | 102 765 99 939 | 4 838 4 736 | 4 566 4 503 | 5 291 5 208 | 7 090 6 977 |
| Aware reduction factor | 0.25 0.75 | 116 902 111 477 | 5 552 4 512 | 5 346 4 255 | 6 186 4 958 | 8 309 6 617 |
| Treatment reduction factor | 0 1 | 114 135 111 185 | 5 032 4 718 | 4 810 4 493 | 5 542 5 143 | 7 473 6 898 |

*Liver-related deaths averted in comparison with status quo were represented in the sensitivity analysis. Note that number of liver-related deaths averted under the base case may not necessarily reside in the range obtained by low and high parameter values since the overall number of liver-related deaths under status quo also changed for each parameter in the sensitivity analysis. Therefore, trends for low and high values should be evaluated independently. Lastly, results could be influenced by first-order uncertainty that could result in inconsistent trends. Under status quo, 160 inmates were treated regardless of their fibrosis stages or prisons’ HCV prevalence. Strategy 1 prioritizes inmates by their fibrosis stages (fibrosis scores F4, F3, F2, F1, and F0) with a treatment capacity of 2,000/year, irrespective of the prison or region. Strategy 2 prioritizes prisons by their HCV prevalence with a treatment capacity of 2,000/year, irrespective of fibrosis stages. Strategy 3 considers unlimited capacity. In Strategies 1–3, only those sentenced with more than six months are eligible for treatment. Strategy 4 considers unlimited treatment capacity and assumed everyone, irrespective of their sentence length, is eligible for treatment.

Abbreviations: HCV, hepatitis C virus; METAVIR, meta-analysis of histologic data in viral hepatitis; F0-F4, METAVIR fibrosis score; SVR, sustained virologic response.

**Table S6.** Results of one-way sensitivity analysis showing new infections averted.

| **Parameter** | **Value  (Low/ High)** | **New Infections** | **New Infections Averted*** | | | |
| --- | --- | --- | --- | --- | --- | --- |
|  |  |  | **(Compared with Status Quo)** | | | |
|  |  | **Status Quo** | **Strategy 1** | **Strategy 2** | **Strategy 3** | **Strategy 4** |
| **Base Case** |  | **84 191** | **6 477** | **6 525** | **7 638** | **10 205** |
| **HCV-Associated Agent Characteristics** | |  |  |  |  |  |
| Awareness: Community | 0.05 0.55 | 110 988 72 067 | 7 127 5 963 | 7 133 5 767 | 8 729 6 954 | 11 763 9 159 |
| Awareness: Prisons | 0.7 0.9 | 85 088 84 435 | 6 437 6 536 | 6 494 6 512 | 7 634 7 730 | 10 143 10 216 |
| Treatment experience: Inmates | 0.131 0.331 | 83 870 84 227 | 6 227 6 515 | 6 401 6 386 | 7 452 7 679 | 9 977 10 038 |
| HCV transmission probability | 0.000085 0.000425 | 17 416 253 207 | 890 22 339 | 886 22 111 | 1 061 27 077 | 1 395 37 652 |
| Self-clearance probability | 0.23 0.28 | 85 825 81 750 | 6 724 6 253 | 6 622 6 150 | 7 916 7 369 | 10 486 9 596 |
| Newborn infection rate | 0.0061% 0.018% | 84 128 84 195 | 6 577 6 425 | 6 487 6 353 | 7 758 7 743 | 10 226 10 091 |
| **Transition Probabilities** |  |  |  |  |  |  |
| F3 to hepatocellular carcinoma (annual) | 0.003 0.014 | 85 352 82 918 | 6 555 6 460 | 6 570 6 352 | 7 784 7 575 | 10 368 10 070 |
| Compensated cirrhosis (F4) to decompensated cirrhosis (annual) | 0.01 0.079 | 88 974 76 026 | 6 714 6 118 | 6 827 5 808 | 7 997 7 088 | 10 664 9 356 |
| Compensated cirrhosis (F4) to hepatocellular carcinoma (annual) | 0.01 0.079 | 90 217 73 545 | 6 970 5 786 | 6 904 5 796 | 8 335 6 642 | 10 886 8 866 |
| SVR after cirrhosis to decompensated cirrhosis (annual) | 0.002 0.036 | 84 191 85 009 | 6 477 6 502 | 6 525 6 399 | 7 638 7 726 | 10 205 10 073 |
| SVR after cirrhosis to hepatocellular carcinoma (annual) | 0.002 0.013 | 84 112 84 301 | 6 596 6 512 | 6 519 6 469 | 7 819 7 756 | 10 297 10 146 |
| Decompensated cirrhosis to hepatocellular carcinoma (annual) | 0.03 0.083 | 84 765 83 913 | 6 673 6 575 | 6 617 6 455 | 7 921 7 563 | 10 296 10 058 |
| Decompensated cirrhosis to liver transplant (annual) | 0.01 0.062 | 83 931 84 845 | 6 506 6 709 | 6 392 6 560 | 7 653 7 917 | 10 134 10 392 |
| Decompensated cirrhosis (first year) to liver-related death (annual) | 0.065 0.19 | 84 740 84 023 | 6 615 6 619 | 6 572 6 439 | 7 808 7 612 | 10 404 10 155 |
| Decompensated cirrhosis (subsequent year) to liver-related death (annual) | 0.065 0.19 | 84 970 83 079 | 6 509 6 401 | 6 447 6 299 | 7 796 7 526 | 10 262 9 948 |
| Hepatocellular carcinoma to liver transplant (annual) | 0 0.14 | 83 613 85 217 | 6 498 6 484 | 6 386 6 390 | 7 646 7 519 | 10 064 10 159 |
| Hepatocellular carcinoma to liver-related death (annual) | 0.33 0.86 | 85 023 82 723 | 6 710 6 406 | 6 591 6 427 | 7 818 7 582 | 10 393 10 175 |
| Liver transplant (first year) to liver-related death (annual) | 0.06 0.42 | 84 203 83 676 | 6 570 6 466 | 6 473 6 386 | 7 712 7 556 | 10 183 10 121 |
| Liver transplant (subsequent year) to liver-related death (annual) | 0.024 0.11 | 84 482 83 728 | 6 607 6 536 | 6 451 6 283 | 7 713 7 537 | 10 296 10 246 |
| **Agent's Behavior** |  |  |  |  |  |  |
| F0 HCV diagnosis probability | 0.02787 0.04606 | 78 388 75 325 | 6 406 6 035 | 6 323 6 143 | 7 409 7 223 | 9 832 9 505 |
| F1 HCV diagnosis probability | 0.02236 0.03702 | 78 400 75 315 | 6 242 6 134 | 6 178 6 031 | 7 367 7 108 | 9 783 9 504 |
| F2 HCV diagnosis probability | 0.03179 0.05246 | 79 289 76 988 | 6 424 6 394 | 6 347 6 368 | 7 463 7 484 | 9 913 9 981 |
| F3 HCV diagnosis probability | 0.03471 0.05724 | 80 915 79 128 | 6 344 6 340 | 6 379 6 301 | 7 465 7 446 | 9 921 9 909 |
| F4 HCV diagnosis probability | 0.12439 0.19926 | 75 182 72 969 | 6 190 6 135 | 6 103 6 047 | 7 349 7 114 | 9 689 9 339 |
| Aware reduction factor | 0.25 0.75 | 102 243 67 680 | 10 794 2 836 | 10 650 2 875 | 12 681 3 465 | 16 930 4 654 |
| Treatment reduction factor | 0 1 | 84 191 71 304 | 6 477 5 500 | 6 525 5 387 | 7 638 6 380 | 10 205 8 442 |

New infections averted in comparison with status quo were represented in the sensitivity analysis. Note that number of new infections averted under the base case may not necessarily reside in the range obtained by low and high parameter values since the overall number of new infections under status quo also changed for each parameter in the sensitivity analysis. Therefore, trends for low and high values should be evaluated independently. Lastly, results could be influenced by first-order uncertainty that could result in inconsistent trends.

Under status quo, 160 inmates were treated regardless of their fibrosis stages or prisons’ HCV prevalence. Strategy 1 prioritizes inmates by their fibrosis stages (fibrosis scores F4, F3, F2, F1, and F0) with a treatment capacity of 2,000/year, irrespective of the prison or region. Strategy 2 prioritizes prisons by their HCV prevalence with a treatment capacity of 2,000/year, irrespective of fibrosis stages. Strategy 3 considers unlimited capacity. In Strategies 1–3, only those sentenced with more than six months are eligible for treatment. Strategy 4 considers unlimited treatment capacity and assumed everyone, irrespective of their sentence length, is eligible for treatment.

Abbreviations: HCV, hepatitis C virus; METAVIR, meta-analysis of histologic data in viral hepatitis; F0-F4, METAVIR fibrosis score; SVR, sustained virologic response.

**Table S7** Results of one-way sensitivity analysis showing probability of cost effectiveness of each scenario.

| **Parameter** | **Value  (Low/ High)** | **Probability of cost effectiveness (%)**  **out of 100 runs** | | | | |
| --- | --- | --- | --- | --- | --- | --- |
|  |  |  |  |  |  |  |
|  |  | **Status quo** | **Strategy 1** | **Strategy 2** | **Strategy 3** | **Strategy 4** |
| **HCV-Associated Agent Characteristics** |  |  |  |  |  |  |
| Awareness: Community | 0.05 0.55 | 0 0 | 0 0 | 0 0 | 0 3 | 100 97 |
| Awareness: Prisons | 0.7 0.9 | 0 0 | 0 0 | 0 0 | 9 5 | 91 95 |
| Treatment experience: Inmates | 0.131 0.331 | 0 0 | 0 0 | 0 0 | 2 4 | 98 96 |
| HCV transmission probability | 0.000085 0.000425 | 0 0 | 0 0 | 0 0 | 16 3 | 84 97 |
| Self-clearance probability | 0.23 0.28 | 0 0 | 0 0 | 0 0 | 8 7 | 92 93 |
| Newborn infection rate | 0.0061% 0.018% | 0 0 | 0 0 | 0 0 | 3 7 | 97 93 |
| **Health-Related Quality-of-Life Parameters** |  |  |  |  |  |  |
| METAVIR score F0, F1 | 0.99 0.837 | 0 0 | 0 2 | 0 0 | 0 30 | 100 62 |
| METAVIR score F2, F3 | 0.99 0.837 | 0 0 | 0 0 | 0 0 | 0 35 | 100 65 |
| Compensated cirrhosis (METAVIR score F4) | 0.99 0.81 | 0 0 | 0 0 | 0 0 | 0 26 | 100 74 |
| Decompensated cirrhosis | 0.88 0.72 | 0 0 | 0 0 | 0 0 | 5 12 | 95 88 |
| Hepatocellular carcinoma | 0.869 0.711 | 0 0 | 0 0 | 0 0 | 6 10 | 94 90 |
| First-year post liver transplant | 0.924 0.756 | 0 0 | 0 0 | 0 0 | 6 8 | 94 92 |
| Post SVR (F0-F1) | 0.99 0.9 | 0 0 | 0 0 | 0 0 | 6 6 | 94 94 |
| Post SVR (F2-F4) | 0.99 0.837 | 0 0 | 0 0 | 0 0 | 6 7 | 94 93 |
| **Costs (in Euros)** |  |  |  |  |  |  |
| **Health State Cost** | |  |  |  |  |  |
| F0, F1 | 182.5 547.5 | 0 0 | 0 0 | 0 0 | 6 6 | 94 94 |
| F2, F3 | 140  420 | 0 0 | 0 0 | 0 0 | 6 6 | 94 94 |
| Compensated cirrhosis (F4) | 280  840 | 0 0 | 0 0 | 0 0 | 6 6 | 94 94 |
| Decompensated cirrhosis | 1 140 3 420 | 0 0 | 0 0 | 0 0 | 6 6 | 94 94 |
| Hepatocellular carcinoma | 3 350 10 050 | 0 0 | 0 0 | 0 0 | 6 6 | 94 94 |
| Liver transplant, first year | 52 000 156 000 | 0 0 | 0 0 | 0 0 | 6 6 | 94 94 |
| Liver transplant, subsequent year | 8 900 26 700 | 0 0 | 0 0 | 0 0 | 6 6 | 94 94 |
| **Testing and Treatment Costs** | |  |  |  |  |  |
| HCV ELISA test (anti-HCV antibody test) | 1.5 4.5 | 0 0 | 0 0 | 0 0 | 6 6 | 94 94 |
| Quantitative HCV RNA | 20 60 | 0 0 | 0 0 | 0 0 | 6 6 | 94 94 |
| Fibroscan test | 30 90 | 0 0 | 0 0 | 0 0 | 6 6 | 94 94 |
| HCV treatment | 9 933 29 799 | 0 0 | 0 0 | 0 0 | 6 30 | 94 70 |
| **Transition Probabilities** |  |  |  |  |  |  |
| F3 to hepatocellular carcinoma (annual) | 0.003 0.014 | 0 0 | 0 0 | 0 0 | 6 5 | 94 95 |
| Compensated cirrhosis (F4) to decompensated cirrhosis (annual) | 0.01 0.079 | 0 0 | 0 0 | 0 0 | 7 4 | 93 96 |
| Compensated cirrhosis (F4) to hepatocellular carcinoma (annual) | 0.01 0.079 | 0 0 | 0 0 | 0 0 | 10 7 | 90 93 |
| SVR after cirrhosis to decompensated cirrhosis (annual) | 0.002 0.036 | 0 0 | 0 0 | 0 0 | 6 10 | 94 90 |
| SVR after cirrhosis to hepatocellular carcinoma (annual) | 0.002 0.013 | 0 0 | 0 0 | 0 0 | 4 4 | 96 96 |
| Decompensated cirrhosis to hepatocellular carcinoma (annual) | 0.03 0.083 | 0 0 | 0 0 | 0 0 | 5 2 | 95 98 |
| Decompensated cirrhosis to liver transplant (annual) | 0.01 0.062 | 0 0 | 0 0 | 0 0 | 2 4 | 98 96 |
| Decompensated cirrhosis (first year) to liver-related death (annual) | 0.065 0.19 | 0 0 | 0 0 | 0 0 | 5 7 | 95 93 |
| Decompensated cirrhosis (subsequent year) to liver-related death (annual) | 0.065 0.19 | 0 0 | 0 0 | 0 0 | 7 6 | 93 94 |
| Hepatocellular carcinoma to liver transplant (annual) | 0 0.14 | 0 0 | 0 0 | 0 0 | 5 9 | 95 91 |
| Hepatocellular carcinoma to liver-related death (annual) | 0.33 0.86 | 0 0 | 0 0 | 0 0 | 5 7 | 95 93 |
| Liver transplant (first year) to liver-related death (annual) | 0.06 0.42 | 0 0 | 0 0 | 0 0 | 2 3 | 98 97 |
| Liver transplant (subsequent year) to liver-related death (annual) | 0.024 0.11 | 0 0 | 0 0 | 0 0 | 8 7 | 92 93 |
| **Agent's Behavior** |  |  |  |  |  |  |
| F0 HCV diagnosis probability | 0.02787 0.04606 | 0 0 | 0 0 | 0 0 | 4 6 | 96 94 |
| F1 HCV diagnosis probability | 0.02236 0.03702 | 0 0 | 0 0 | 0 0 | 2 4 | 98 96 |
| F2 HCV diagnosis probability | 0.03179 0.05246 | 0 0 | 0 0 | 0 0 | 2 7 | 98 93 |
| F3 HCV diagnosis probability | 0.03471 0.05724 | 0 0 | 0 0 | 0 0 | 3 2 | 97 98 |
| F4 HCV diagnosis probability | 0.12439 0.19926 | 0 0 | 0 0 | 0 0 | 7 8 | 93 92 |
| Aware reduction factor | 0.25 0.75 | 0 0 | 0 0 | 0 0 | 7 6 | 93 94 |
| Treatment reduction factor | 0 1 | 0 0 | 0 0 | 0 0 | 6 7 | 94 93 |

*We conducted sensitivity-analysis by running the model 100 times for each parameter value and presented the probability of each strategy being cost-effective using €21,000 willingness to pay threshold in Spain. Due to possible first-order uncertainty the results could lead to inconsistent trends such as extended dominance. To prevent misinterpretation of this trends, instead of presenting ICER values; we presented the likelihood of each strategy being cost-effective, as commonly done in generating cost-effectiveness acceptability curves.

Under status quo, 160 inmates were treated regardless of their fibrosis stages or prisons’ HCV prevalence. Strategy 1 prioritizes inmates by their fibrosis stages (fibrosis scores F4, F3, F2, F1, and F0) with a treatment capacity of 2,000/year, irrespective of the prison or region. Strategy 2 prioritizes prisons by their HCV prevalence with a treatment capacity of 2,000/year, irrespective of fibrosis stages. Strategy 3 considers unlimited capacity. In Strategies 1–3, only those sentenced with more than six months are eligible for treatment. Strategy 4 considers unlimited treatment capacity and assumed everyone, irrespective of their sentence length, is eligible for treatment.

Abbreviations: HCV, hepatitis C virus; METAVIR, meta-analysis of histologic data in viral hepatitis; F0-F4, METAVIR fibrosis score; ELISA, enzyme-linked immunosorbant analysis; SVR, sustained virologic response. Under scenarios


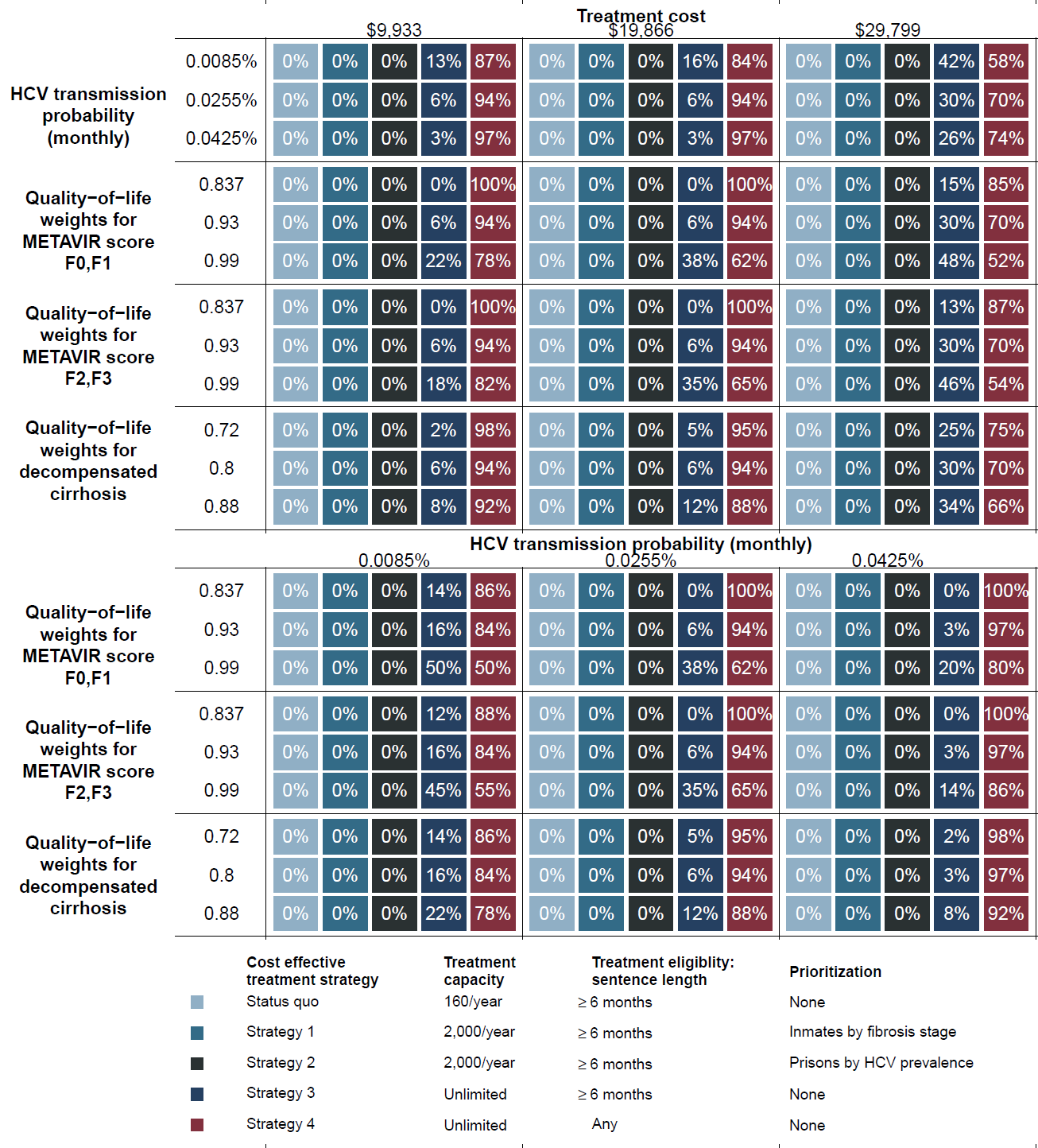
*We conducted sensitivity-analysis by running the model 100 times for each parameter value and presented the probability of each strategy being cost-effective using €21,000 willingness to pay threshold in Spain. Due to possible first-order uncertainty the results could lead to inconsistent trends such as extended dominance. To prevent misinterpretation of this trends, instead of presenting ICER values; we presented the likelihood of each strategy being cost-effective, as commonly done in generating cost-effectiveness acceptability curves.

Abbreviations: HCV, hepatitis C virus; METAVIR, meta-analysis of histologic data in viral hepatitis; F0-F4, METAVIR fibrosis score.

**Figure S2** Results of two-way sensitivity analysis on select parameters showing probability of cost effectiveness of each scenario.

**REFERENCES**

1. He, T. *et al.* Prevention of hepatitis C by screening and treatment in US prisons. *Annals of internal medicine* **164**, 84-92 (2016).

2. Stroustrup, B. *The C++ programming language*. (Pearson Education India, 2000).

3. Arnold, K., Gosling, J. & Holmes, D. *The Java programming language*. Vol. 2 (Addison-wesley Reading, 1996).

4. *National Institute of Statistics*, <<http://www.ine.es/>> (2019).

5. Vlahov, D. *et al.* Mortality risk among recent-onset injection drug users in five US Cities. *Subst Use Misuse* **43**, 413-428 (2008).

6. Spaulding, A. C. *et al.* Prisoner survival inside and outside of the institution: implications for health-care planning. *American journal of epidemiology* **173**, 479-487, doi:10.1093/aje/kwq422 (2011).

7. Susana Lierena, C. C., Silvia Alvarez, Angel Estebanez, Miguel Mateo, Jose Ramon Pallas, Santiago Echevarria, Ana Saez, Jesus Aguero,Natalia Chueca, Raul Pellon, Juan Crespo del Pozo, Antoni Cuadrado, Federico Garcia, Javier Crespo. A program of Testing and Treat Intended to Eliminate Hepatitis C in a Prison: The JAILFREE-C study. *Abstract ID:916, AASLD Liver Meeting, 2016*.

8. Crespo, J., Llerena, S., Cobo, C. & Cabezas, J. Is HCV elimination possible in prison? *Revista espanola de sanidad penitenciaria* **19**, 70-73 (2018).

9. Crespo J et.al. Is elimination of HCV infection from penitentiary centers possible? The Jailfree-C trial: a sustained “test and treat” program in inmates. Manuscript submitted to the Am J Gastroenterology

10. Da Silva A, Planella R, Sastre A, et al. Variables asociadas a la discontinuación del tratamiento en presos tratados de hepatitis C crónica en Cataluña en el periodo 2002-2016. Qué ha cambiado con el uso de los antivirales de acción directa? GEHEP congress 2017, Poster 070.

11. Tellez F, Carrillo CF, Ramirez D, et al. Eficacia de la terapia con AAD en los centros penitenciarios de Puerto I, II y III.GEHEP congress 2017; Poster O42.

12. Daivozadeh G, Nieto MA, Campo E, et al. Análisis descriptivo de la situación de la hepatitis C en los centros penitenciarios de Puerto I, II y III. GEHEP congress 2016; Oral 15

13. Peinado M, Cabanes Y, Nardini C, et al. Tratamiento directamente observado con antivirales de acción directa del virus de la Hepatitis C en pacientes del centro penitenciario de Valencia-Picassent. GEHEP congress 2016, Poster 62.

14. Marco A, Da Silva, Planella R, et al. Análisis del uso de antivirales de acción directa en la práctica clínica para el tratamiento de la Hepatitis C Crónica en las prisiones de Cataluña. GEHEP congress 2016; Poster 52

15. Fernandez F, Matilla AM, Gijon P, et al. Sofosbuvir / ledipasvir in Spanish prison population with chronic hepatitis C. EASL congress 2016; Poster SAT-154

16. *Prevalence of HIV and HCV infection in penitentiary institutions,* , <<http://www.institucionpenitenciaria.es/web/export/sites/default/datos/descargables/saludpublica/Prevalencia_VIH_y_VHC_en_IIPP_2016.pdf>> (2015).

17. Cardoso, A. C. *et al.* Impact of peginterferon and ribavirin therapy on hepatocellular carcinoma: incidence and survival in hepatitis C patients with advanced fibrosis. *Journal of Hepatology* **52**, 652-657 (2010).

18. Lok, A. S. *et al.* Incidence of hepatocellular carcinoma and associated risk factors in hepatitis C-related advanced liver disease. *Gastroenterology* **136**, 138-148, doi:10.1053/j.gastro.2008.09.014 (2009).

19. Benvegnu, L. *et al.* Evidence for an association between the aetiology of cirrhosis and pattern of hepatocellular carcinoma development. *Gut* **48**, 110-115 (2001).

20. Fattovich, G. *et al.* Morbidity and mortality in compensated cirrhosis type C: a retrospective follow-up study of 384 patients. *Gastroenterology* **112**, 463-472 (1997).

21. Gentilini, P. *et al.* Long course and prognostic factors of virus-induced cirrhosis of the liver. *The American journal of gastroenterology* **92**, 66-72 (1997).

22. Sangiovanni, A. *et al.* The natural history of compensated cirrhosis due to hepatitis C virus: A 17 year cohort study of 214 patients. *Hepatology* **43**, 1303-1310 (2006).

23. Serfaty, L. *et al.* Determinants of outcome of compensated hepatitis C virus-related cirrhosis. *Hepatology* **27**, 1435-1440 (1998).

24. Bruno, S. *et al.* Hepatitis C virus genotypes and risk of hepatocellular carcinoma in cirrhosis: a prospective study. *Hepatology* **25**, 754-758, doi:10.1002/hep.510250344 (1997).

25. Tateyama, M. *et al.* Alpha-fetoprotein above normal levels as a risk factor for the development of hepatocellular carcinoma in patients infected with hepatitis C virus. *Journal of gastroenterology*, 1-9 (2011).

26. Tsukuma, H. *et al.* Risk factors for hepatocellular carcinoma among patients with chronic liver disease. *New England journal of medicine* **328**, 1797 (1993).

27. Yoshida, H. *et al.* Interferon therapy reduces the risk for hepatocellular carcinoma: national surveillance program of cirrhotic and noncirrhotic patients with chronic hepatitis C in Japan. *Annals of internal medicine* **131**, 174 (1999).

28. Planas, R. *et al.* Natural history of decompensated hepatitis C virus-related cirrhosis. A study of 200 patients. *Journal of Hepatology* **40**, 823-830 (2004).

29. Thuluvath, P. *et al.* Liver transplantation in the United States, 1999–2008. *American Journal of Transplantation* **10**, 1003-1019 (2010).

30. Davis, G. L., Alter, M. J., El-Serag, H., Poynard, T. & Jennings, L. W. Aging of hepatitis C virus (HCV)-infected persons in the United States: a multiple cohort model of HCV prevalence and disease progression. *Gastroenterology* **138**, 513-521, doi:10.1053/j.gastro.2009.09.067 (2010).

31. Lang, K., Danchenko, N., Gondek, K., Shah, S. & Thompson, D. The burden of illness associated with hepatocellular carcinoma in the United States. *J Hepatol* **50**, 89-99 (2009).

32. Saab, S., Hunt, D. R., Stone, M. A., McClune, A. & Tong, M. J. Timing of hepatitis C antiviral therapy in patients with advanced liver disease: A decision analysis model. *Liver Transpl* **16**, 748-759 (2010).

33. Wolfe, R., Roys, E. & Merion, R. Trends in organ donation and transplantation in the United States, 1999–2008. *American Journal of Transplantation* **10**, 961-972 (2010).
